# Characterization in *Helicobacter pylori* of a Nickel Transporter Essential for Colonization That Was Acquired during Evolution by Gastric *Helicobacter* Species

**DOI:** 10.1371/journal.ppat.1006018

**Published:** 2016-12-06

**Authors:** Frédéric Fischer, Marie Robbe-Saule, Evelyne Turlin, Francesco Mancuso, Valérie Michel, Pierre Richaud, Frédéric J. Veyrier, Hilde De Reuse, Daniel Vinella

**Affiliations:** 1 Institut Pasteur, Département de Microbiologie, Unité Pathogenèse de *Helicobacter*, ERL CNRS 3526, Paris, FRANCE; 2 CEA, DRF, BIAM SBVME and CNRS, UMR 7265, Saint-Paul-lez-Durance, Aix Marseille Université, Marseille, FRANCE; 3 INRS-Institut Armand-Frappier, Bacterial Symbionts Evolution, Laval, Quebec, CANADA; University of California Davis School of Medicine, UNITED STATES

## Abstract

Metal acquisition is crucial for all cells and for the virulence of many bacterial pathogens. In particular, nickel is a virulence determinant for the human gastric pathogen *Helicobacter pylori* as it is the cofactor of two enzymes essential for *in vivo* colonization, urease and a [NiFe] hydrogenase. To import nickel despite its scarcity in the human body, *H*. *pylori* requires efficient uptake mechanisms that are only partially defined. Indeed, alternative ways of nickel entry were predicted to exist in addition to the well-described NixA permease. Using a genetic screen, we identified an ABC transporter, that we designated NiuBDE, as a novel *H*. *pylori* nickel transport system. Unmarked mutants carrying deletions of *nixA*, *niuD* and/or *niuB*, were constructed and used to measure (i) tolerance to toxic nickel exposure, (ii) intracellular nickel content by ICP-OES, (iii) transport of radioactive nickel and (iv) expression of a reporter gene controlled by nickel concentration. We demonstrated that NiuBDE and NixA function separately and are the sole nickel transporters in *H*. *pylori*. NiuBDE, but not NixA, also transports cobalt and bismuth, a metal currently used in *H*. *pylori* eradication therapy. Both NiuBDE and NixA participate in nickel-dependent urease activation at pH 5 and survival under acidic conditions mimicking those encountered in the stomach. However, only NiuBDE is able to carry out this activity at neutral pH and is essential for colonization of the mouse stomach. Phylogenomic analyses indicated that both *nixA* and *niuBDE* genes have been acquired via horizontal gene transfer by the last common ancestor of the gastric *Helicobacter* species. Our work highlights the importance of this evolutionary event for the emergence of *Helicobacter* gastric species that are adapted to the hostile environment of the stomach where the capacity of *Helicobacter* to import nickel and thereby activate urease needs to be optimized.

## Author Summary

*Helicobacter pylori* is a bacterium that persistently colonizes the stomach of half of the human population worldwide. Infection by *H*. *pylori* is associated with gastritis, peptic ulcer disease and adenocarcinoma. To resist gastric acidity and proliferate in the stomach, *H*. *pylori* possesses an urease, this enzyme contains a nickel-metallocenter at its active site. Thus, the capacity of *H*. *pylori* to infect its host relies on important nickel supply despite its scarcity in the human body. To import nickel, *H*. *pylori* requires efficient mechanisms that were partially defined. In the present study, we identified a novel *H*. *pylori* nickel transport system that we designated NiuBDE. NiuBDE was directly demonstrated to import nickel into the bacterium. NiuBDE and the previously described NixA permease are the two sole nickel transporters in *H*. *pylori*. Urease activation by nickel depends on the NiuBDE and NixA systems and NiuBDE is essential for colonization of a mouse model by *H*. *pylori*. We found that NiuBDE and NixA have been acquired during evolution by *H*. *pylori* and other gastric-*Helicobacter* species. Thus, acquisition of nickel transporters by gastric *Helicobacter* was a key event for the emergence of one of the most successful bacterial pathogens, *H*. *pylori*.

## Introduction

Transition metals are essential elements for living organisms because they are involved in many enzymatic reactions and physiological processes. For bacteria, metals have been shown to be essential for survival under various conditions including those faced during infection [1]. Tremendous efforts have been devoted to deciphering the mechanisms of iron uptake, trafficking and intracellular homeostasis control. Much less information is available on the acquisition and homeostasis of nickel, which is nevertheless an essential element for many bacteria [2]. Indeed it is the cofactor of at least nine enzymes involved in diverse cellular processes such as energy and nitrogen metabolism/detoxification and virulence [3], including [NiFe] hydrogenase, urease, Ni-SOD and CO dehydrogenase [4,5].

Nickel is a virulence determinant for *Helicobacter pylori*, a pathogen that colonizes the stomach of about half of the human population worldwide and causes a variety of gastric pathologies ranging from gastritis to adenocarcinoma [6,7] [8]. In order to successfully colonize the stomach, which is a highly acidic environment, *H*. *pylori* depends on urease [9]. Urease requires nickel to catalyze hydrolysis of urea into carbon dioxide and ammonia [10]. These buffering compounds are essential to maintain the pH close to neutrality in the bacterial cytoplasm [11,12]. Urease accounts for up to 6% of the soluble cellular proteins [13]. In *H*. *pylori*, nickel is also required for the activity of a second enzyme, a [NiFe] hydrogenase shown to be important for colonization, presumably because it provides an alternative respiratory pathway, allowing *H*. *pylori* to use molecular hydrogen as an energy source [14]. Since nickel is required for urease and hydrogenase activities, *H*. *pylori* has to acquire sufficient nickel ions, while preventing the toxic effects of non-physiological intracellular metal concentrations that would interfere with incorporation of other metals in essential proteins. Thus, *H*. *pylori* strictly controls intracellular nickel concentration with multiple mechanisms of sensing, transport and protection. Several proteins involved in these processes have been described. These include NikR, a nickel responsive pleiotropic transcriptional regulator [15–17], CznABC a metal efflux pump [18] and also three Histidine-rich proteins Hpn, Hpn-2 and HspA that are found only in the *Helicobacter* genus [19–22]. Hpn and HspA are involved in sequestration of nickel ions and the corresponding mutants are highly sensitive to nickel exposure [21,22]. In addition, the Hpn/Hpn-2 and HspA nickel-binding proteins contribute to the production of active urease and hydrogenase, respectively [22–24].

The concentration of nickel ions in the human body is low (1–11 nM) [25]. Consequently *H*. *pylori* requires highly specific importers of Ni(II) ions. These mechanisms are only partially identified. In Gram-negative bacteria, energized transport of metabolites such as iron-siderophore complexes through the outer membrane (OM) relies on the TonB machinery and on TonB dependent-transporters (TBDTs). We previously described FrpB4, the first TonB-dependent nickel transport system across a bacterial OM [26,27]. The activity of FrpB4 is acid-induced and its expression is repressed by nickel via NikR. This allows *H*. *pylori* to optimize nickel uptake and nickel-dependent activation of urease under conditions where ammonia production needs to be maximal. By analogy with siderophores, it is possible that a nickelophore, *i*.*e*. a small organic chelator of nickel, is required for transport by this TBDT. Studies highlighted that a nickelophore is required for nickel binding to the periplasmic NikA protein from *Escherichia coli* [28,29] or for nickel uptake by the *Staphylococcus aureus* Cnt transporter [30].

Transport of nickel ions across the cytoplasmic membrane is generally accomplished either by multiple-components, ATP-binding-cassette-containing transporters or by single component secondary transporters of the NiCoT family (nickel/cobalt transporter [31]) that use the physiochemical gradient of the cytoplasmic membrane as an energy source. The ABC-type NikABCDE transporter, first described in *E*. *coli* [32], is composed of NikA, a periplasmic binding protein, NikBC, two integral membrane components, and NikDE, two ATPases that energize substrate translocation through ATP hydrolysis. Another nickel ABC transporter has been identified in *Campylobacter jejuni*, that is composed of NikZYXWV [33,34]. In *S*. *aureus*, nickel is taken up by two ABC transporters NikABCDE and CntABCDF in addition to the NixA permease [35–37].

In *H*. *pylori*, the only nickel transport system across the cytoplasmic membrane that has been reported so far is NixA, a permease of the NiCoT family [38,39], whose expression is repressed by nickel [17,40]. Inactivation of NixA renders *H*. *pylori* more resistant to nickel overload [41]. However, NixA deficient mutants were reported to retain urease activity [42] and are still able to colonize the mouse model at levels comparable to that of a wild type strain [43]. Several other proteins have therefore been proposed to participate in nickel uptake [44,45], but the role of none of them was confirmed.

The *Helicobacter* genus is composed of two subgroups, the enterohepatic species (EH) that infect the liver or gastrointestinal tract of mammals and some birds and a small group of gastric *Helicobacter* species (including *H*. *pylori*) [46]. Only few information is available on the presence of nickel transporters in the non-*pylori* gastric *Helicobacter* and EH *Helicobacter* species. In *Helicobacter mustelae* that colonizes ferrets, NikH, a TBDT different from FrpB4 and a CeuE/FecDE ABC transport system were proposed to be involved in nickel uptake as the corresponding mutants are affected in urease activity and intracellular nickel content [47]. In the mouse-colonizing *Helicobacter hepaticus*, NikR regulates the expression of genes encoding homologues of the *E*. *coli* nickel ABC transporter (NikABCDE) and of the *H*. *mustelae* NikH [48].

In the present work, we applied a genetic screen to search for additional nickel transporters in *H*. *pylori*. We found that an ABC transporter, that we designated NiuBDE, transports nickel separately from NixA. In addition, we provide evidence that NiuBDE, but not NixA, also transports cobalt and bismuth, a metal currently used in *H*. *pylori* eradication therapy in humans. Phylogenomic analyses revealed that both transport systems have been acquired by the last common ancestor of the gastric *Helicobacter* species via horizontal gene transfer. Finally, we demonstrated that, contrary to NixA, the NiuBDE transporter is indispensable for colonization of the mouse model by *H*. *pylori*. We conclude that acquisition of nickel transporters has been an important evolutionary event to allow *Helicobacter* to adapt to colonization of the hostile gastric environment.

## Results

### Selection and characterization of *H*. *pylori* mutants resistant to high Ni(II) concentrations

Nickel toxicity depends on import into the cell. Therefore, in order to search for new Ni(II)-uptake mechanism(s), we applied a genetic screen to select for *H*. *pylori* mutants able to survive exposure to high Ni(II) concentrations. A genomic library constructed by N.R Salama [49], in which *H*. *pylori* genes were randomly inactivated *in vitro* by the insertion of a transposable element conferring chloramphenicol resistance, was used to transform *H*. *pylori* strain G27 [50] and the isogenic *Δhpn* mutant that is more sensitive to nickel [22]. We first established the nickel concentrations at which the plating efficiencies of strains G27 and G27 *Δhpn* were strongly affected. This corresponded to 1 mM for wild type G27 strain and only 500 μM for the G27 *Δhpn* mutant. After transformation with the library, several mutants resistant to chloramphenicol and able to grow on plates in the presence of lethal concentrations of nickel were obtained in four independent selection experiments. Chromosomal DNA of these mutants was extracted and used to localize the transposon insertion sites by nested PCR [49]. Two independent insertions conferring nickel resistance in both the G27 and G27 Δ*hpn* backgrounds were mapped inside the *hpG27_842* open reading frame.

The *hpG27_842* gene has been annotated as *fecD* because of its sequence homology with the *E*. *coli fecD* gene whose product is involved in ferric citrate transport. *E*. *coli* citrate-mediated iron transport system is expressed by five genes grouped into a single operon, designated *fecABCDE* [51]. *fecA and fecB* encode a TBDT outer membrane protein and a periplasmic protein, respectively. The *fecC* gene and its paralogue *fecD* encode cytoplasmic membrane proteins, *fecE* codes for an ATP-binding protein associated with the cytoplasmic membrane. It has been shown that after FecA-mediated TonB-dependent transport of iron(III) dicitrate across the outer membrane, FecBCDE transport iron across the cytoplasmic membrane. In *H*. *pylori* strains, the annotated operon is restricted to two genes, *fecD* followed by *fecE*. In *H*. *pylori* G27 strain, the *fecDE* promoter region is a possible NikR target as it was pulled out in a NikR ChIP-Seq experiment [52]. The selection of *fecD* insertions during our screen prompted us to examine whether this protein could be involved in nickel transport across the inner membrane. Since our experiments confirmed this activity, this gene was renamed, for sake of clarity, *niuD* for nickel uptake protein D and the downstream gene, annotated *fecE*, was renamed *niuE*.

### NiuD and NixA inactivation increases *H*. *pylori* tolerance to high nickel concentrations

**Fig 1:**
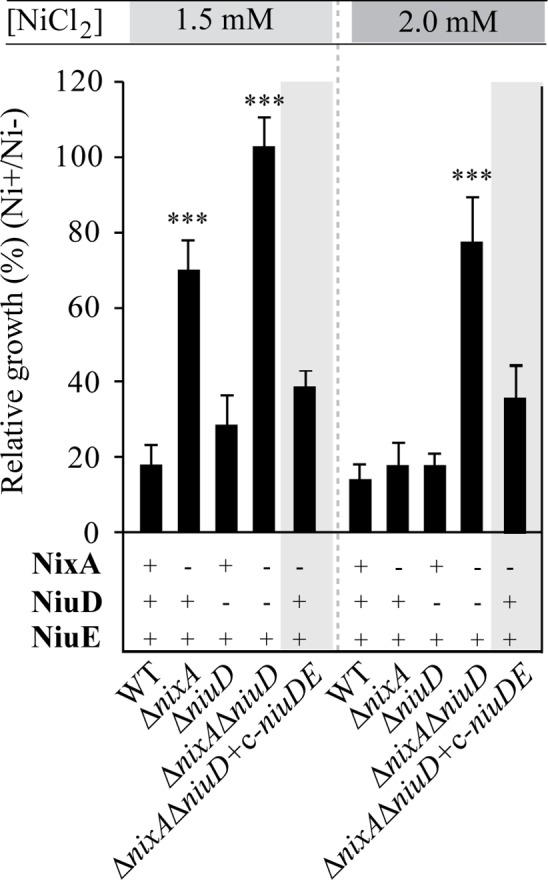
NiuD and NixA mediate *H*. *pylori* sensitivity to high nickel concentrations. Effect of 1.5 and 2 mM NiCl_2_ on growth of *H*. *pylori* B128-S wild type strain, isogenic mutants and complemented strains. The results are presented as % of growth in the presence of nickel relative to growth without nickel after 24h incubation. The data correspond to the mean value of three independent experiments. Error bars represent the standard deviation. *** indicates that the mean value is significantly different from that of the wild type strain (*P* ≤ 0.001).

Using a previously described method [53], we constructed unmarked deletions mutants of *niuD* (*hpB8_663*) in the B128-S and B128-S Δ*hpn* strains, (a genetic background that we previously characterized [22], S1 and S2 Tables). Tolerance of these mutants to nickel exposure was evaluated at neutral pH by following growth inhibition in liquid medium. Surprisingly, we observed that the *niuD* deletion did not significantly increase nickel tolerance in the B128-S (Fig 1) or B128-S Δ*hpn* background (S1 Fig). We then examined the effect of these mutations in combination with a deletion of the gene encoding NixA, the previously identified nickel permease. The Δ*nixA* mutation increased nickel tolerance in both *hpn* ^*+*^ and *Δhpn* backgrounds but only at the lowest nickel concentration tested (Figs 1 and S1). We found that growth of the Δ*nixA* Δ*niuD* mutants was insensitive to nickel exposure up to 2 mM. Thus, the Δ*nixA* Δ*niuD* mutants were significantly more tolerant to exposure to high nickel concentrations than the single Δ*nixA* mutants in both *hpn* ^+^ and Δ*hpn* backgrounds. To test complementation, the *niuDE* genes were cloned under the control of the *ureI* promoter in the pIRC(P_*ureI*_) vector [54] [22] and introduced at a neutral position on the chromosome of our different mutants (designated c-*niuDE*). The Δ*nixA ΔniuD*/c-*niuDE* strain thus constructed recovered nickel tolerance levels comparable to that of the parental Δ*nixA* mutant (Fig 1).

Altogether, these results strongly suggest that NiuD is part of a novel nickel transport system that is distinct from NixA.

### NiuD and NixA inactivation results in reduced intracellular nickel content

**Fig 2:**
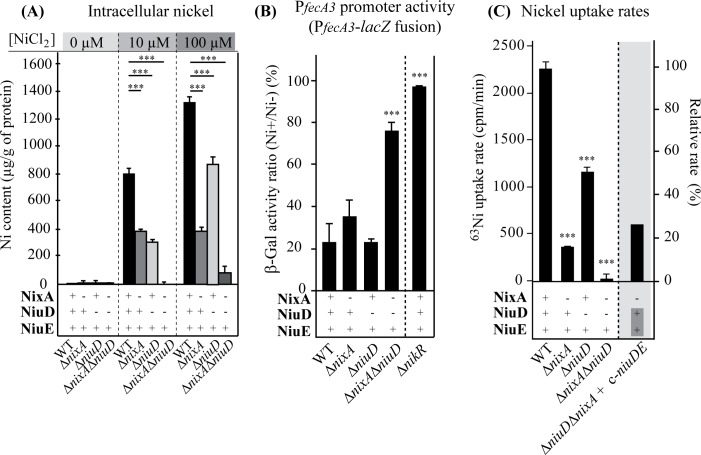
NiuD and NixA control the intracellular nickel content and uptake in *H*. *pylori*. Panel A: Nickel amounts measured by Inductively Coupled Plasma Optical Emission Spectrometry (ICP-OES) and expressed as μg of nickel.g^-1^ of protein. Strains were grown either without added nickel, with 10 μM or with 100 μM NiCl_2_. Panel B: ß-galactosidase activity expressed by the P_*fecA3*_::*lacZ* reporter fusion in wild type and mutant strains. The expression of the fusion decreases in a NikR-dependent manner with increasing intracellular nickel concentration. ß-galactosidase activities are presented as the ratio of activity measured in strains grown in the presence of 200 μM nickel versus without nickel, expressed in percentages. Panel C: Measurements of radioactive nickel uptake rates in wild type and mutant strains expressed in cpm/min. On the right side scale, the uptake rates were normalized with respect to the value measured for the wild type strain. Complementation with the *niuDE* operon inserted at a neutral locus on the chromosome is designated c-*niuDE* (c- stands for “chromosomally inserted”). In these different experiments, the data correspond to the mean value of three independent experiments and error bars represent the standard deviation. *** indicates that the mean value is significantly different (*P* ≤ 0.001) from that of the wild type strain.

To evaluate the role of NiuD in nickel uptake, we measured the total intracellular nickel content of our collection of mutants grown in the presence of various nickel concentrations by Inductively-coupled plasma optical emission spectrometry (ICP-OES) as previously described [22] (Fig 2A). No nickel could be detected in strains grown without nickel supplementation. In the wild type strain grown with 10 and 100 μM NiCl_2_, intracellular nickel content reached 800 and 1,300 μg/g prot, respectively. In the Δ*nixA* mutant, nickel content reached a constant value that was, under both conditions, 2-fold (at 10 μM NiCl_2_) and 3-fold (at 100 μM NiCl_2_) lower than that of the wild type strain. Similarly, the Δ*niuD* mutant presented a reduced nickel content equivalent to that of the Δ*nixA* mutant in the presence of 10 μM nickel. When grown with 100 μM nickel, intracellular nickel content of the *ΔniuD* mutant increased to a level that was still about 1.6-fold lower than that of the wild type strain under the same conditions. Importantly, nickel content of the Δ*nixA* Δ*niuD* double mutant was below the detection limit when the bacteria were exposed to 10 μM NiCl_2_ and reduced by more than 25-fold with 100 μM nickel. Similar results were obtained in the Δ*hpn* genetic background (S2 Fig). These data indicate that the Δ*niuD* and Δ*nixA* mutations have additive effects on intracellular nickel accumulation.

Another way to evaluate the intracellular nickel concentration is to measure the expression of a reporter gene that is under the control of the nickel-responsive transcriptional regulator of *H*. *pylori*, NikR. The well-characterized P_*fecA3*_ promoter, which is repressed by NikR in response to nickel [17,55], was used to construct a pILL2157 derivative plasmid carrying a P_*fecA3*_::*lacZ* fusion. This construct was transformed into strain B128-S and its isogenic mutants Δ*nixA*, Δ*niuD*, Δ*nixA* Δ*niuD* and *ΔnikR* (Fig 2B). As expected, ß-galactosidase activity of the *ΔnikR* mutant was strongly enhanced and insensitive to nickel addition, validating the reporter system. ß-galactosidase activity measured in the wild type strain and the mutants was about 5,000 miller units in non-supplemented medium. Addition of 200 μM NiCl_2_ to the wild type strain did not cause significant growth inhibition and resulted in a rapid decrease in ß-galactosidase activity observable as soon as 4 hours after nickel addition. After 24 hours growth, the activity of the fusion was decreased about 5-fold in both the wild type strain and the Δ*niuD* mutant and about 2.5-fold in the Δ*nixA* mutant as compared to the control condition without added nickel (Fig 2B). Nickel addition to the Δ*niuD* Δ*nixA* mutant only resulted in a slight 1.3-fold reduction of ß-galactosidase activity. These results are consistent with a diminished NikR-dependent repression of the reporter fusion as a consequence of minimal intracellular nickel accumulation in this double mutant.

Comparing the phenotypes of the mutants with the wild type strain, we found a good correlation between diminished nickel sensitivity, a decrease in total nickel content and derepression of the P_*fecA3*_ promoter. Our results show that inactivation of both the *nixA* and *niuD* genes resulted in a massive defect in accumulation of nickel into the cells, strongly indicating that NiuD and NixA are nickel uptake systems.

### NiuD and NixA are involved in two separate nickel uptake pathways in *H*. *pylori*

To obtain definitive proof of the involvement of NiuD in nickel transport, we measured the uptake rates of radioactive ^63^Ni(II) in the wild type and mutant strains in the presence of 10 μM total nickel at pH 5 (Fig 2C). As previously reported, radioactive ^63^Ni(II) uptake is not measurable at pH 7 as it is, under the test conditions, presumably limited by the acid-activated FrpB4 OM transporter [26]. The Δ*nixA* mutant was dramatically affected with a nickel uptake rate reaching only 17% of that of the wild type, suggesting that NixA is a major contributor in nickel uptake under these conditions.

In the Δ*niuD* mutant, the uptake rates were 51.7% of that of the wild type. Most importantly, the Δ*nixA* Δ*niuD* mutant was completely deficient in nickel transport, with an uptake rate of 5%, similar to the background of non-specific nickel binding on filters in control experiments (Fig 2C). Complementation of the Δ*nixA* Δ*niuD* mutant with an ectopic chromosomal copy of the *niuDE* operon (c-*niuDE*) resulted in complete restoration of the Niu-dependent nickel transport, with an uptake rate close to that measured with the Δ*nixA* mutant. Together, these results demonstrate that NixA and NiuD are involved in two distinct nickel uptake systems and strongly suggest that no other nickel transport systems exist in the *H*. *pylori* cytoplasmic membrane.

### NiuB1 and NiuB2 are the periplasmic binding proteins of the ABC nickel uptake system

**Fig 3:**
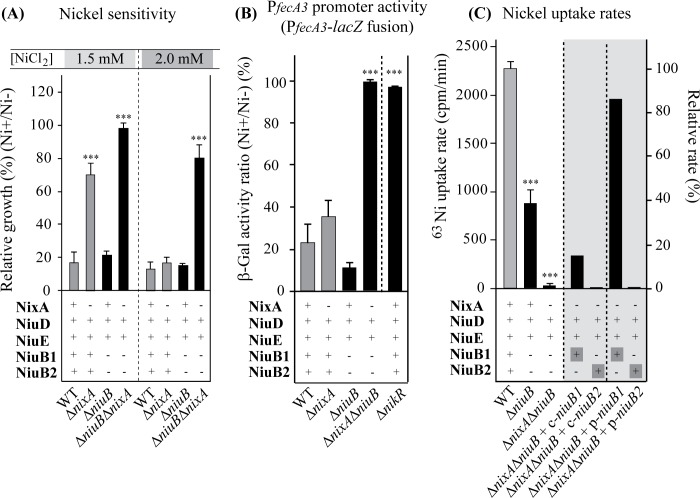
NiuB1 and NiuB2 control the nickel sensitivity and uptake in *H*. *pylori*. Panel A: Effect of nickel on growth of *H*. *pylori* B128-S wild type strain and isogenic mutants. *ΔniuB* corresponds to a *ΔniuB1-ΔniuB2* double mutant. Results are presented as in Fig 1. Panel B: ß-galactosidase activity of the P_*fecA3*_::*lacZ* reporter fusion in different backgrounds presented as in Fig 2. Panel C: Measurements of radioactive nickel uptake rates in wild type and mutant strains. On the right scale, the uptake rates were normalized with respect to the value measured for the wild type strain. The mutant strains were either complemented by c-*niuB1* or c-*niuB2* (c- for chromosomally inserted) inserted at a neutral site on the chromosome under the control of the P_*ureI*_ promoter or by p-*niuB1* and p-*niuB2* (p- for inserted on a plasmid) expressed from derivatives of plasmid pILL2157 under the control of an IPTG-inducible P_*ureI*_ promoter in the presence of IPTG. In these different experiments, the data correspond to the mean value of three independent experiments and error bars represent the standard deviation. *** indicates that the mean value is significantly different (*P* ≤ 0.001) from that of the wild type strain.

Uptake ABC transporters function with high-affinity solute binding proteins that bind the substrate in the periplasm and allow its recognition by the inner membrane transporter proteins. In *H*. *pylori*, a gene annotated as *ceuE* encodes a periplasmic protein that was a good candidate for this function. Although it was annotated as an iron-binding protein, recent publications showed that the *ceuE* gene is regulated by NikR in response to nickel [52] and that the CeuE protein, whose structure has been solved, binds nickel [56]. Some *H*. *pylori* strains such as B128 possess two consecutive and paralogous genes annotated *ceuE1* (*hpB8_1657*) and *ceuE3* (*hpHPB8_1658*) that share 85% identity, which we renamed *niuB1* and *niuB2*. An unmarked deletion of the two genes, referred to as Δ*niuB*, was constructed. We found that this deletion alone did not influence nickel tolerance in the wild type B128-S strain (Fig 3A). However, as observed with the Δ*niuD* mutation, deletion of *niuB* increased nickel tolerance in the Δ*nixA* genetic background (Fig 3A). In addition, while expression of the P_*fecA3*_::*lacZ* fusion was slightly affected in the Δ*niuB* mutant as compared to the wild type strain, strong derepression was observed in the Δ*nixA* Δ*niuB* double mutant. This phenotype, indicative of a total absence of nickel uptake, was similar to that of the Δ*nixA* Δ*niuD* mutant (Figs 2B and 3B). Thus, the behavior of the *ΔniuB* mutants fully parallels that of the Δ*niuD* mutants and points to the participation of NiuB in nickel transport together with the NiuD and NiuE proteins.

As mentioned before, some *H*. *pylori* strains harbor two NiuB parologues. To analyze the individual contribution of the two paralogues, we cloned the *niuB1* and *niuB2* genes separately and transformed them into the Δ*nixA* Δ*niuB* mutant. Both *niuB1* or *niuB2* genes expressed either from the chromosome (designated c-) or from a plasmid under the control of an IPTG inducible promoter (designated p-) restored nickel sensitivity to levels comparable to those of the Δ*nixA* mutant (see below). Radioactive nickel uptake rates measured in the Δ*niuB* was 38% that of the wild type strain and less than 5% for the Δ*nixA* Δ*niuB* mutant (Fig 3C). This again paralleled the results obtained for the Δ*niuD* and Δ*nixA* Δ*niuD* mutants, further confirming that NiuB and NiuD are indeed part of the same transport system (Fig 3C). Intriguingly, when expressed from the chromosome (c-*niuB1* and c-*niuB2* in Fig 3C), NiuB1 but not NiuB2 was able to restore ^63^Ni(II) uptake in the Δ*nixA* Δ*niuB* mutant to the level of the genetically equivalent Δ*nixA* mutant (compare Fig 3C to Fig 2C). In the Δ*nixA* Δ*niuB* double mutant, IPTG-induced overexpression of NiuB1 from plasmid pILL2157 (p-*niuB1*) resulted in a strong increase of nickel uptake rate reaching 95% of wild type level. In contrast, no restoration of nickel uptake was observed with NiuB2 even when overexpressed under the same conditions (p-*niuB2*). Thus, NiuB1 appears to be the major periplasmic component for nickel uptake by the NiuBDE ABC transport system.

### Role of NiuD, NiuB1-2 and NixA in nickel-dependent urease activation

**Fig 4:**
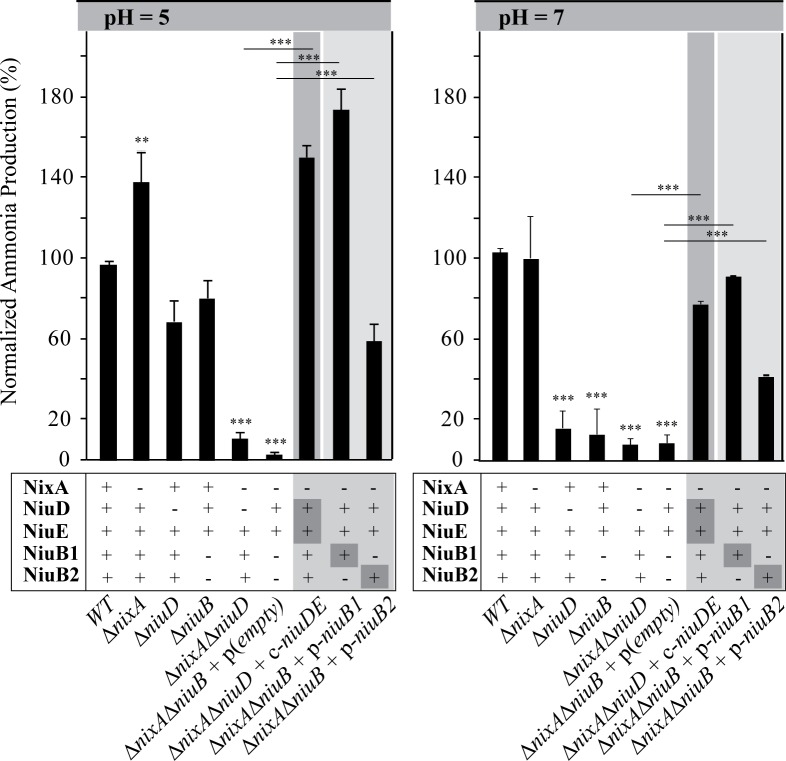
Role of NixA and NiuBDE in urease activity of *H*. *pylori* at pH 5 and 7. Urease activity was measured on whole cells of the different mutants strains by measuring ammonia production. For each condition, ammonia production is expressed as a percentage of the wild type strain put at 100%. These data indicate that NiuBDE functions at both pH 5 and 7, while NixA is mainly active at pH 5. *ΔniuB* corresponds to a *ΔniuB1-ΔniuB2* double mutant. The mutant strains were either complemented by c-*niuDE* (c- for chromosome) inserted at a neutral site on the chromosome under the control of the P_*ureI*_ promoter or by p-*niuB1* and p-*niuB2* (p- for plasmid) expressed from derivatives of plasmid pILL2157 under the control of an IPTG-inducible P_*ureI*_ promoter in the presence of IPTG. The data correspond to the mean value of three independent experiments and error bars represent the standard deviation. *** and ** indicate that the mean value is significantly different from that of the wild type strain (*P* ≤ 0.001 and *P* ≤ 0.01, respectively). For the complemented strains, *ΔnixA* Δ*niuD* + c-*niuDE* or *ΔnixA* Δ*niuB* p-*niuB1/2*, the *P* values correspond to comparison with the corresponding parental *ΔnixA ΔniuD* and *ΔnixA* Δ*niuB* mutants, respectively.

We then evaluated the role of the two nickel uptake systems in the activation of the nickel-dependent urease in strains incubated in BBß medium, which contains 0.2 μM nickel [40]. Urease activity was tested in intact live cells exposed to pH 5 or pH 7 by measuring ammonia produced from urea hydrolysis in wild-type and mutant strains as previously described [57] (Fig 4). We first found that urease activity did not decrease in the Δ*nixA* mutant at neutral pH and rather slightly increased under pH 5 acidic conditions (140%) compared to the parental wild type strain. At pH 5, urease-dependent ammonia production of the *niuD* and *niuB* mutants were similar and only slightly affected (70% of the wild type strain). In contrast, at pH 7, these two mutants presented a strong decrease in urease activity, reaching only 15% of the wild type value and suggesting that the remaining NixA transporter is not supporting nickel uptake under this condition. This suggests that NiuD activity is independent of the pH, while NixA is only efficient under acidic pH conditions. Finally, at both pH values ammonia production of the *ΔnixA* Δ*niuD* and Δ*nixA* Δ*niuB* double mutants was dramatically decreased to a negligible level under both conditions (<10% of the wild type values). This indicates that in these mutants, there is a strong defect in nickel delivery to urease as a consequence of deficient nickel transport.

When the *niuDE* operon was expressed ectopically in the *ΔnixA* Δ*niuD* double mutant (c-*niuDE*), urease activity was restored to levels similar to that of the Δ*nixA* single mutant. Plasmidic expression of either NiuB1 or NiuB2 (p-*niuB1*, p-*niuB2*) in the Δ*nixA* Δ*niuB* mutant restored urease activity under the two pH conditions. However, only expression of NiuB1 could complement the double mutant to wild type levels, while NiuB2 only partially restored urease activity. This again argues in favor of NiuB1 being more efficient in nickel transport than its NiuB2 paralogue.

Overall, these results indicate that *H*. *pylori* possesses two nickel uptake machineries, NiuBDE and NixA, that function separately from each other to deliver nickel to the major virulence factor urease. Contrary to NiuBDE that functions at both pH 5 and 7, NixA is much more active under acidic conditions than at neutral pH.

### Both NiuBDE and NixA are required for survival to acid exposure

**Fig 5:**
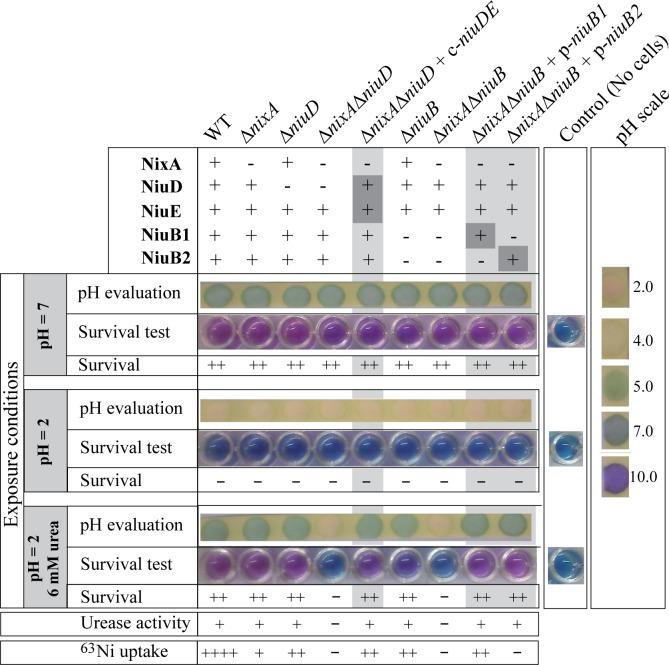
NixA and NiuBDE are necessary for resistance to extreme acidity in the presence of urea. Wild type strains and mutants were exposed during 40 min at pH 2, without or with 6 mM urea. *ΔniuB* corresponds to a *ΔniuB1-ΔniuB2* double mutant. Controls were performed at pH 7 without urea. Survival of the bacteria was assessed using an Alamar blue-based test, in which a pink staining reveals metabolically active cells, while a blue staining means that cells are metabolically inactive. The pink staining is proportional to the amount of metabolically active cells. The pH was evaluated for each strain and results were compared to control cells (exposed at pH 7) and to a pH-scale (right side of the figure). A reminder of the results obtained in other experiments for urease activity and ^63^Ni(II) uptake is given at the bottom of the figure for comparison.

We further examined the respective importance of the two nickel uptake systems in the *H*. *pylori* survival capacity under extreme acidic conditions, mimicking those encountered in the gastric environment (Fig 5). Wild-type B128-S strain and mutants were tested for their ability to enhance the pH of a highly acidic buffer (PBS at pH 2, as in [58]) through urea breakdown and ammonia synthesis. In addition, survival of the cells was assessed with an Alamar blue-based viability test [59]. Without added urea, no strain survived a 40 minutes-long exposure at pH 2 and the pH of the medium remained acidic. In the presence of 6 mM exogenous urea, wild type strain and the Δ*nixA*, Δ*niuD* and Δ*niuB* single mutants survived and their ammonia production resulted in pH increase to values close to neutrality, in line with their urease activity measurements (Fig 5). In contrast, the Δ*nixA* Δ*niuD* and Δ*nixA* Δ*niuB* double mutants were unable to increase the pH and, as a result, did not survive high acidity exposure. This phenotype was restored to wild type when an ectopic *niuDE* copy (c-*niuDE*) was introduced on the chromosome of the Δ*nixA* Δ*niuD* mutant. Interestingly, both *niuB1* and *niuB2* genes expressed from plasmids (p-*niuB1* and p-*niuB2*) complemented the Δ*nixA* Δ*niuB* mutant to wild type phenotype. This demonstrates that under these conditions, both periplasmic NiuB1 and NiuB2 proteins are functional to mediate sufficient nickel entry within the cells. Thus, under conditions mimicking those of the stomach, NixA or NiuBDE is sufficient for survival to extreme acidity.

### Metal specificity of the NiuBDE transporter

To determine whether the novel *H*. *pylori* NiuBDE transport system is involved in uptake of metals other than nickel, sensitivity tests were carried out with our collection of mutants. The effect of addition of zinc, iron, copper or manganese on growth of the Δ*niuD*, Δ*nixA* and Δ*nixA* Δ*niuD* mutants did not differ from that on the parental wild type strain. This suggests that the NiuBDE and NixA transport systems do not participate in uptake of these metals.

**Fig 6:**
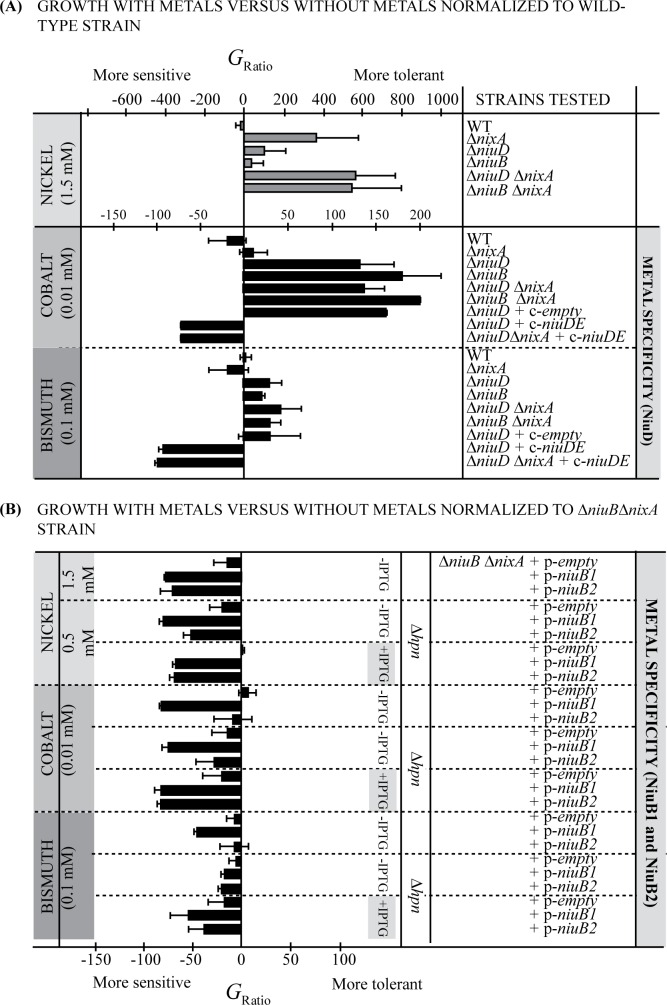
Sensitivity to cobalt and bismuth of wild type strain and isogenic mutants. Effect of NiCl_2,_ CoCl_2_ and bismuth subcitrate potassium on growth of *H*. *pylori* B128-S wild type strain, isogenic mutants and complemented strains. *ΔniuB* corresponds to a *ΔniuB1-ΔniuB2* double mutant. Strains were grown 24 h in the presence or absence of the metal examined. Results are presented as a Growth ratio G_Ratio_ = 100x[(OD_+metal_/OD_-metal_)_test_−(OD_+metal_/OD_-metal_)_ref_] / (OD_+metal_/OD_-metal_)_ref_ allowing to normalize the growth ratio of the test strain compared to the growth ratio of the reference strain. The reference is the wild type strain on panel A and the *ΔnixA ΔniuB* mutant on panel B. Bars correspond to the G_Ratio_ values. This representation allows to visualize the results in such a way that bars on the left relative to the vertical axis correspond to G_Ratio_ values of strains that are more sensitive to the metal than the wild type strain (negative values) and those on the right side to G_Ratio_ values of strains that are more tolerant (positive values) to the metal. The results are presented together with those with nickel for comparison. The data correspond to the mean value of three independent experiments and error bars represent the standard deviation.

We then performed tests with cobalt and bismuth (Fig 6). The sensitivity of the Δ*nixA* mutant to cobalt did not significantly differ from that of the parental strain (in agreement with [41]). In contrast, the Δ*niuD*, Δ*niuB* and Δ*nixA* Δ*niuD* mutants presented the same strong increase in tolerance to 0.01 mM cobalt exposure. Complementation of the Δ*niuD* and Δ*nixA* Δ*niuD* mutants with c-*niuDE* rendered the strains more sensitive to cobalt than the wild type strain, indicating that more cobalt accumulated under these conditions. These results suggest that NiuBDE, but not NixA, is involved in cobalt uptake in *H*. *pylori*.

For bismuth exposure, no significant difference was observed with the *ΔnixA* mutant while tolerance of the Δ*niuD*, Δ*niuB* and Δ*nixA* Δ*niuD* mutants was enhanced, although this phenotype was less marked than with cobalt or nickel. However, the increased sensitivity of strains overexpressing *niuDE* strongly suggests that uptake of bismuth is mediated by the NiuBDE transporter and not by the NixA permease.

Similar experiments were carried out to determine which, if any, NiuB periplasmic protein is required for bismuth and cobalt uptake. We tested the Δ*nixA* Δ*niuB* and Δ*nixA* Δ*niuB Δhpn* mutants complemented with a plasmid expressing either *niuB1* or *niuB2* under the control of an IPTG-inducible promoter (p-*niuB1*, p-*niuB2*). Without IPTG, only the Δ*nixA* Δ*niuB* and Δ*nixA* Δ*niuB Δhpn* mutants complemented with *niuB1* recovered wild type sensitivity to cobalt and bismuth, suggesting that NiuB1 plays a major role in uptake of these metals under these conditions. However, when *niuB1* or *niuB2* expression was induced by IPTG, cobalt and bismuth sensitivity was restored to level of the *ΔnixA Δhpn* mutant, indicating that both NiuB1 and NiuB2 can function in cobalt and bismuth uptake.

We concluded that cobalt and bismuth are taken up by the NiuBDE transporter in *H*. *pylori*. The existence of an additional transporter for these metals can however not be excluded.

### NiuD and NiuB1-2 but not NixA are essential for colonization of the mouse model

**Fig 7:**
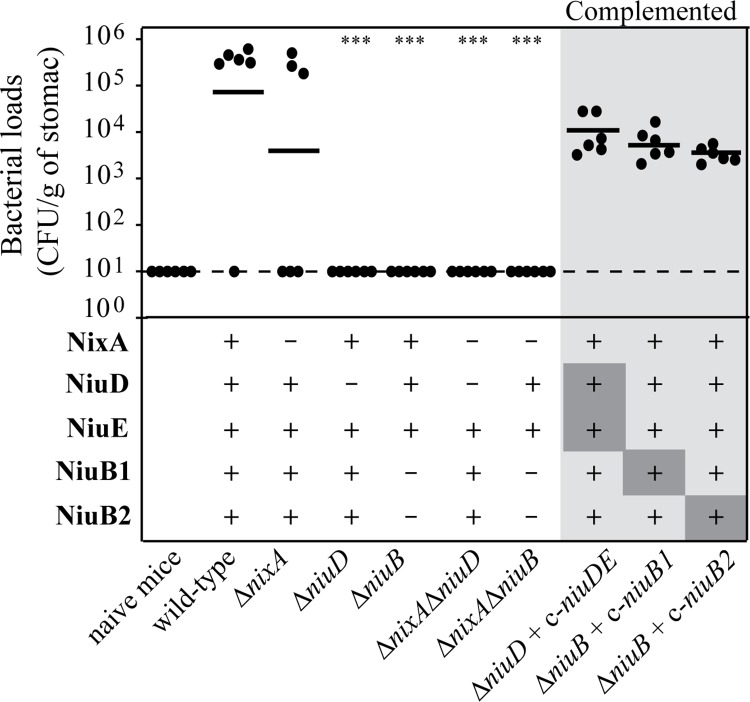
NiuD and NiuB are required for mouse colonization by *H*. *pylori* strain SS1. Each point corresponds to the colonization load for one mouse one month after infection with the strain indicated below. Horizontal bars represent the geometric means of the colonization load for the wild type, each mutant and the chromosomally complemented mutants (designated c-). *ΔniuB* corresponds to a *ΔniuB1-ΔniuB2* double mutant. The results presented correspond to a representative experiment out of two. The detection limit is shown by a dashed horizontal line. *** indicates that the geometric value is significantly different (*P* ≤ 0.001) from that of the wild type strain.

The role of NiuD and NiuB1-NiuB2 during gastric colonization was evaluated using the mouse model. *H*. *pylori* SS1 mutants carrying complete deletions of the *niuD* or *niuB1 niuB2* (*ΔniuB*) genes and/or *nixA* genes were constructed. The *ΔniuD* mutant was recomplemented on the chromosome with a *niuDE* copy (c-*niuDE*). The *ΔniuB* mutant was recomplemented with either *niuB1* or *niuB2* (c-*niuB1*, c-*niuB2*). Each strain was orogastrically inoculated in six NMRI mice. One month later, colonization was assessed by quantitative cultures of stomach homogenates (Fig 7). The geometric mean of the colonization loads of mice infected with the Δ*nixA* mutant was only slightly below that of the wild type strain, indeed half of the mouse were colonized at the level of the parental strain. In contrast, the Δ*niuD* and Δ*niuB* mutants were completely deficient in their capacity to colonize the mouse stomach, indicative of their major role in pathogenicity. Each of the complemented strain, SS1 Δ*niuD* c-*niuDE*, SS1 Δ*niuB* c-*niuB1* and SS1 Δ*niuB* c-*niuB2* recovered close to wild type capacity to colonize the murine stomach. These results show that NixA is not essential for *in vivo* colonization. In contrast, the NiuBDE system is indispensable for colonization of the mouse stomach and *in vivo* can function with either NiuB1 or NiuB2.

### Distribution and phylogeny of the nickel transporters in the *Helicobacter* species

**Fig 8:**
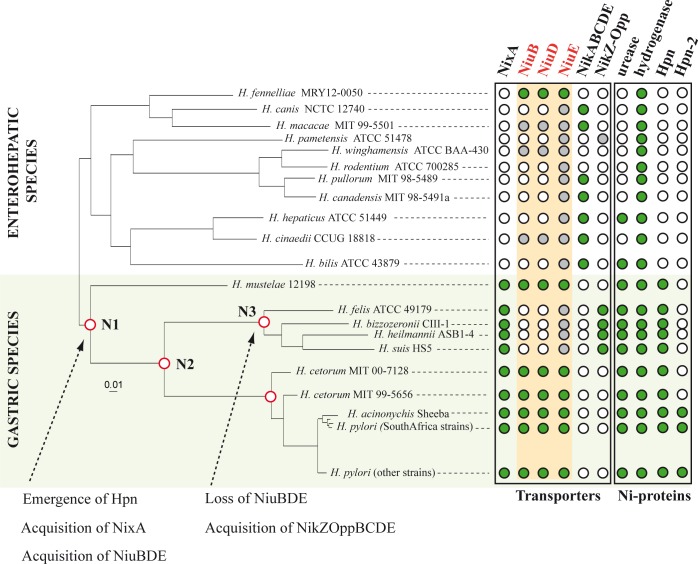
Evolutionary history of *Helicobacter* nickel inner membrane transporters. The core proteome-based neighbor-joining phylogenetic tree of *Helicobacter* species was obtained in a previous study [22]. Enterohepatic (EH) and gastric species are indicated and the latter are highlighted with a light green background. Names of each species are indicated. Important nodes (N1, N2 and N3) are indicated in red. For each strain, the table indicates the presence (green circles) or the absence (white circles) of the proteins indicated: transporters (NixA, NiuBDE, NikABCDE and NikZOppBCDE), Ni-dependent enzymes (urease, hydrogenase) or Ni-binding proteins (Hpn and Hpn-2). Grey circles indicate low identity orthologs that were detected in strains but that have no direct phylogenetical relationship to the proteins studied, according to the maximum likelihood (ML) trees. Arrows and comments describe the evolutionary events that account for the observations and phylogenetical analyses. At node N1, the last common ancestor of gastric species, in which Hpn emerged [22], also acquired NixA and NiuBDE transporters. NiuBDE was vertically inherited by the subsequent gastric species. NiuBDE was then lost in the *heilmannii*-like branch (node N3) and replaced by another putative nickel ABC transporter (NikZOppBCDE, a possible NikZYXWV-like system).

The *Helicobacter* genus is composed of two subgroups, the enterohepatic (EH) species and a small group of gastric *Helicobacter* species including *H*. *pylori* that exclusively colonize the stomach of diverse mammals and depend on urease activity for their multiplication in this niche. The central role of NiuBDE suggested by our work prompted us to establish its distribution in the *Helicobacter* genus, to search for other predicted inner membrane nickel transporters and to compare their distribution. We screened full proteomes of available *Helicobacter* genomes (NCBI) (11 EH species, 8 non-*pylori* gastric *Helicobacter* species and the 434 *H*. *pylori* strains analyzed in [22]). The MycoHIT program [60,61] was used to search for NixA, subunits of NiuBDE, NikABCDE, NikZYXWV, urease (UreB) and hydrogenase (HydA). The identified proteins were then mapped onto a previously-determined phylogenetic tree of *Helicobacter* species [22] (Fig 8). First, we observed that both NixA and NiuBDE are conserved in every *H*. *pylori* strain. As expected, urease is present in all gastric species and in only 5 EH species (S3 Fig). Hydrogenase is ubiquitous. The distribution of NixA and NiuBDE is not correlated with the presence of urease but rather follows the gastric/EH tropism of the *Helicobacter* species. Indeed, NixA is present in every gastric *Helicobacter* species and is not detected in EH species. The picture is more complex for NiuBDE. The 3 subunits were unambiguously detected in 5 gastric species but not in *H*. *felis*, *H*. *bizzozeronnii*, *H*. *suis*, *H*. *heilmannii* (*heilmannii*-like group) and in only one EH species, *H*. *fennelliae*. Instead, 10 EH species out of 14 examined are equipped with a NikABCDE nickel transporter.

Interestingly, in all *heilmannii*-like species tested, a NikZ protein (>60% identity) was detected, its corresponding *nikZ* gene being part of a conserved operon of five genes encoding a predicted Opp-type ABC-transporter (S3 Fig). These gastric *Helicobacter* species probably possess a NikZYXWV-like nickel transporter [33,34], as an alternative to the Niu transporter.

The observation that NixA is restricted to the gastric *Helicobacter* species can either correspond to gene acquisition by these species or gene loss in the EH *Helicobacter* species. To obtain a picture of the evolutionary history of both systems, we established the phylogeny of the NixA, NiuB and NiuD proteins. The maximum likelihood (ML) phylogenetic tree of NixA protein sequences shows that they emerge from within the Firmicutes, suggesting an acquisition of *nixA* via horizontal gene transfer (HGT) from *Paenibacilli* to the last common ancestor (LCA) of the gastric *Helicobacter* species (S4 Fig). ML trees of NiuB and NiuD revealed a patchy taxonomic distribution, indicative of a complex evolutionary history (S5 and S6 Figs). However, NiuB and NiuD from gastric *Helicobacter* species form a clade distinct from other EH species and Epsilonproteobacteria, and closely related to Thermodesulfobacteria and Deltaproteobacteria, together with those from the *H*. *fennelliae* (EH species) and *Campylobacter sputorum*. Importantly, the NiuB and NiuD proteins display very similar evolutionary histories (S7 Fig). Comparison of gene synteny with phylogenies reveals that *niuB* and *niuD* genes were originally in an operon and were likely transferred together from Thermodesulfobacteria to *C*. *sputorum* and then to *H*. *fennelliae* (Epsilonproteobacteria). Then, the *niuBDE* operon would have been transferred further from an EH species related to *H*. *fennelliae* to the LCA of gastric species. This operon would have been vertically inherited in other gastric species, except for *heilmannii*-like species, where the NiuBDE cluster was most likely lost and/or functionally replaced by a NikZYXWV-like system.

Among the gastric *Helicobacter* species, *H*. *pylori* is the only species that presents either one or two copies of NiuB depending on the strain. *niuB1* is always located upstream of *niuB2* and corresponds to the single *niuB* from species having only one copy. The two copies are always in tandem suggesting a recent duplication event.

In conclusion, our phylogenomic analysis revealed that the two nickel transporters of *H*. *pylori* were acquired by HGT in gastric *Helicobacter* species. This acquisition was probably an important step in evolution to optimize the capacity of *Helicobacter* strains to thrive in the stomach.

## Discussion

Pathogenicity of bacteria depends on their capacity to acquire sufficient metals to survive within their hosts. In the case of *H*. *pylori*, nickel is a virulence determinant, since it is a cofactor of urease, which is essential for surviving the acidic environment of the stomach, and therefore for gastric colonization. Acquisition of nickel from the extracellular environment is a major challenge for *H*. *pylori* since its concentration is very low in the human body [25]. Besides an outer membrane nickel transporter (FrpB4) [26], *H*. *pylori* possesses a high affinity nickel permease of the NiCoT family in the inner membrane (NixA) that has been extensively described [38,62]. A *nixA* deletion mutant retains urease activity and is still able to colonize the mouse model [43]. Therefore, it was proposed that another nickel transport system would be functional to fuel the bacterium with this metal.

**Fig 9:**
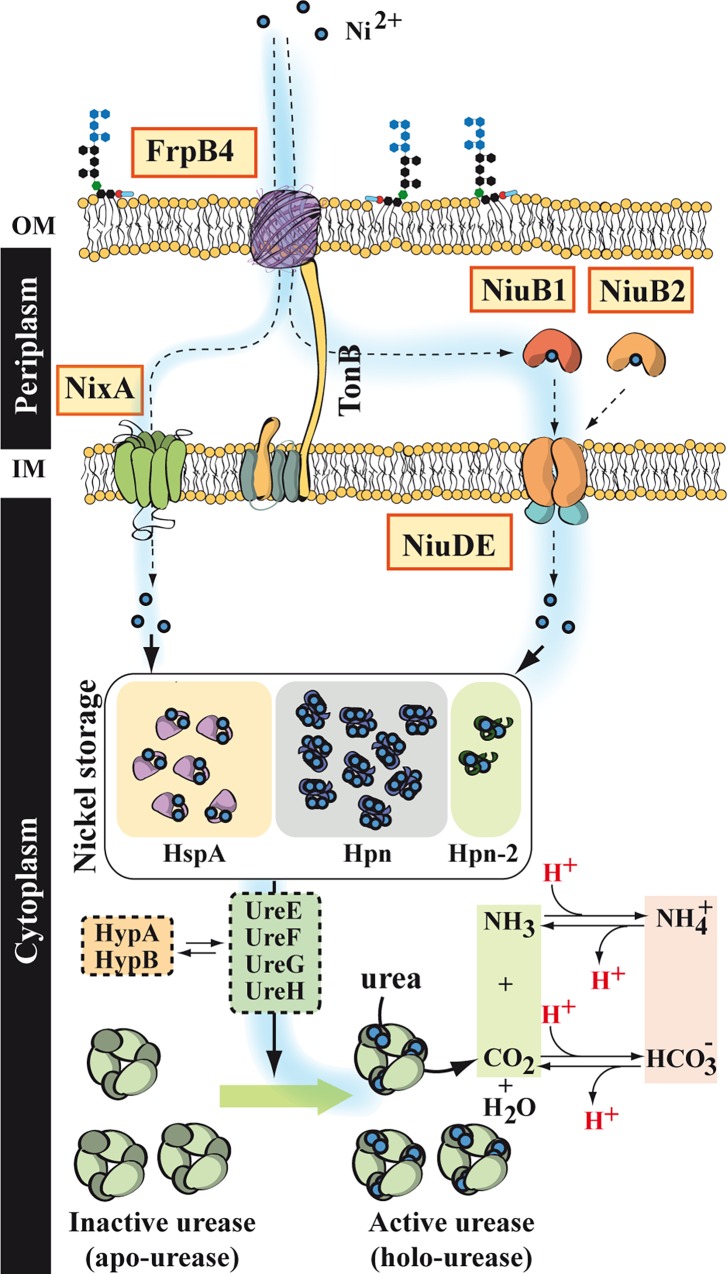
Model for nickel transport in *H*. *pylori*. In *H*. *pylori*, nickel (small blue circles) is transported across the outer membrane by FrpB4 (purple barrel), a TonB-dependent transporter. This uptake activity is most efficient at acidic pH. Once in the periplasm, uptake of nickel through the inner membrane can be performed by the NixA permease (green). Alternatively, nickel can form a Ni(II)-(L-His)_2_ complex, that is specifically recognized by the periplasmic solute binding protein NiuB (orange). In *H*. *pylori* strains with two NiuB paralogs, NiuB1 (dark orange) seems to be the major contributor for nickel uptake, while NiuB2 (light orange) is less efficient. Then, NiuB docks onto the NiuD permease (orange), and nickel is transferred across the inner membrane upon ATP consumption by NiuE (light blue), and delivered to the cytoplasm. There, it is stored by histidine-rich proteins, such as HspA (GroES, purple), Hpn (blue) or Hpn-2 (green) and/or channeled by the UreEFGH machinery toward urease, or by the HypAB machinery towards hydrogenase. The figure presents urease activation through nickel delivery by UreEFGH (to which HypAB also contributes) in the cytoplasm. Ultimately, this pathway results in nickel-dependent urease activation, this enzyme catalyzing urea breakdown into ammonia and carbon dioxide, both contributing to maintaining the intracellular pH close to neutrality and allowing the bacteria to resist acidity.

A genome-wide genetic screen allowed us to identify a novel ABC transporter involved in nickel uptake in *H*. *pylori*. This system, that we named Niu (Nickel Uptake), is composed of three proteins: the periplasmic solute binding protein NiuB, together with NiuD and NiuE that are predicted to act as a permease and ATPase subunit, respectively (for a model of nickel transport in *H*. *pylori*, see Fig 9). The function of the Niu system as a nickel transporter was demonstrated in *H*. *pylori* by using nickel sensitivity assays, measurements of intracellular nickel content, expression of a nickel-responsive reporter gene, and direct measurement of ^63^Ni(II) transport kinetics, in single and double mutants deficient in NixA and/or Niu proteins. All of these experiments demonstrated that Niu functions as a genuine nickel transporter in *H*. *pylori*. The fact that the phenotypes of the *ΔnixA* and *Δniu* mutations are additive strongly suggests that the two systems function independently of each other. Strikingly, mutants in which both NixA and Niu systems are inactivated displayed non-measurable nickel uptake rates and very low to undetectable intracellular nickel levels, indicating that they are most likely the two sole inner membrane nickel importers in *H*. *pylori*.

Transport of other metals in *H*. *pylori* was also indirectly examined by sensitivity tests. Cobalt is a known cofactor of several proteins (e.g., arginase in *H*. *pylori* [63]). We showed that the Niu transporter is additionally involved in cobalt uptake, while the NixA permease is not, although the latter belongs to the NiCoT family. Bismuth, a metal that is not a natural protein cofactor, is a component of the new Pylera medication, in combination with two antibiotics and a proton pump inhibitor [64]. Pylera has proven to be more efficient in eradicating *H*. *pylori* infections than the classical triple therapy but the precise mode of action of bismuth and the nature of its toxicity in *H*. *pylori* remains unclear. Our experiments indicate that NiuBDE, but not NixA, participate in bismuth subcitrate potassium uptake in *H*. *pylori*. Thus, *in vivo* bismuth toxicity in *H*. *pylori* might, at least in part, result from its interference with the essential Niu nickel transporter.

We showed that NiuB constitutes the corresponding high-affinity solute binding protein of the Niu transport system. Genomics reveal that ~50% of the sequenced *H*. *pylori* strains, including the B128 strain, possess two closely related paralogous genes, that we designated *niuB1* and *niuB2*, and that are likely the result of a recent duplication. The structure of the NiuB protein from *H*. *pylori* strain G27, which corresponds to the NiuB1 paralogue, has been determined [56]. This protein belongs to the class III periplasmic substrate-binding protein family and binds nickel as a Ni(II)-(L-His)_2_ complex. Binding of such complexes seem to be a common feature for many periplasmic binding proteins of nickel ABC transporters such as NikA in *E*. *coli* or NikZ in *Campylobacter jejuni* [29,34,65]. Our results reveal that NiuB1 is more efficient than NiuB2 in nickel, cobalt and bismuth uptake. Sequence alignments and comparison of B128 NiuB1 and NiuB2 with NiuB from strain G27 indicate that both proteins share all the structural determinants required for the binding of Ni(II)-(L-His)_2_ complex and for the predicted interaction with NiuD [56] (S8 Fig), and only minor differences. Comparison of the putative binding sites reveals only a few substitutions within the binding pocket that could interfere with or influence ligand binding (S8 Fig). The difference in NiuB1 and NiuB2 efficiencies thus remains elusive and future experiments will be needed to understand the precise contribution of each NiuB and to define whether the paralogues underwent functional subspecialization.

In a previous study, we demonstrated that two essential nickel-binding proteins (Hpn and Hpn-2) emerged exclusively in gastric *Helicobacter* species and postulated that this constituted a decisive evolutionary event to allow *Helicobacter* to colonize the hostile gastric environment [22]. Because nickel is necessary to activate urease and therefore to colonize the stomach, we hypothesized that acquisition of nickel transport systems may have followed a similar pattern. We thus analyzed the distribution of all known inner membrane nickel transporters of *Helicobacter* species, and reconstructed the phylogeny of NixA, NiuB and NiuD. Results clearly show that EH species predominantly possess the NikABCDE ABC transport system, as was demonstrated in *H*. *hepaticus* [48]. The *nixA* gene is specific to the gastric *Helicobacter* species and was likely acquired *via* HGT by the LCA of gastric species probably from species closely related to *Paenibacilli*. NiuBDE was probably acquired by the LCA of gastric species *via* HGT from species related to *H*. *fennelliae*. NiuBDE was then inherited vertically by other gastric species and conserved its function as in *H*. *mustelae*, where the corresponding homolog was shown to contribute to nickel acquisition [66]. Within the *heilmannii*-like branch, NiuBDE is absent, and has been functionally replaced by another transporter, comprising NikZ, suggesting that the common ancestor of *heilmannii*-like species may have acquired this NikZYXWV-like system *via* HGT from Firmicutes (as also suggested in [34]). In all cases, unlike EH species, gastric *Helicobacter* species acquired and retained two different nickel transport machineries, NixA and an ABC transporter.

The genomes of gastric *Helicobacter* species are small (1.6 Mb for *H*. *pylori*) suggesting reduced functional redundancy, which has been documented in the case of *H*. *pylori* [67]. Therefore, the existence of two nickel transporters in these organisms is intriguing. Our results indicate that, in *H*. *pylori*, the two transporters differ in their uptake properties as a function of pH and of nickel concentration. Therefore, they might be involved at different stages of the infection in agreement with the differences that we observed during the *in vivo* experiments. At pH 5, the Δ*nixA* mutant displays a much stronger decrease in ^63^Ni(II) uptake rates than the Δ*niuD* mutant, suggesting that NixA is, kinetically, the major contributor under these conditions (Fig 2). However, since urease activity in single Δ*nixA* or *Δniu* mutants does not decrease when compared to the wild type, we conclude that, at pH 5, accumulation of nickel is not significantly affected (Fig 4). Indeed, as shown in Fig 5, individually NixA and Niu can transport sufficient nickel to activate urease and thereby allow survival of *H*. *pylori* to exposure to highly acidic conditions. In contrast, urease activity of the double mutant is very low confirming that these transporters are the two sole nickel uptake systems in *H*. *pylori*.

At pH 7, ^63^Ni(II) uptake cannot be measured possibly because the outer membrane transporter FrpB4 requires acidity to be activated [26]. Under neutral conditions, *ΔnixA* mutants are more tolerant to nickel overload than Δ*niu* mutants suggesting again a predominant role of NixA in nickel uptake. In the *ΔnixA* mutant, in which only the Niu transport system is active, intracellular nickel content measured by ICP-OES, remains constant when extracellular Ni(II) is increased from 10 to 100 μM. In contrast, nickel content doubles in the *Δniu* mutant in which NixA is the only transporter. Thus, the Niu system may already be saturated at low nickel concentrations in contrast to the situation of the NixA transporter. This saturation is likely the basis of higher tolerance to nickel overload in the *nixA* mutant (Fig 1). At pH 7, deletion of the genes encoding the Niu system strongly affects urease activity, while the *ΔnixA* strain displays wild type activity. This suggests that, under these conditions of low nickel availability, NixA is poorly efficient in fueling urease with nickel. We conclude that NixA is required for rapid nickel acquisition at acidic pH, while the Niu system is active under both neutral and acidic conditions even at low nickel concentrations (such as those in the urease activity test). Given that FrpB4 is acid-activated [26], we propose that under neutral conditions, less nickel enters the periplasm thereby providing an advantage to the Niu ABC transporter over the NixA permease, since it is equipped with a high affinity periplasmic binding protein, NiuB.

The *in vivo* function of the two *H*. *pylori* nickel transporters was evaluated by measuring their importance in colonization of a mouse model. The Niu transporter was found to be strictly essential *in vivo*. The *ΔnixA* mutant reproductively presented a bimodal colonization profile, with about half of the mice that were not colonized and the other half that presented normal colonization. This profile is consistent with a bottleneck event in the first steps of colonization, as suggested by previous results reporting that a *nixA* mutant alone can colonize but is outcompeted by a wild type strain during mixed colonization [43]. The non-essentially of the NixA transporter in the mouse model seems surprising given its conservation in all gastric *Helicobacter*. However, our data suggest that NixA and Niu have different transport properties, the Niu system being more adaptable and capable of acquiring nickel at low concentrations under moderately acidic or neutral conditions like those encountered at its persistent colonization site, the epithelial cell surface. It could also be that during *in vivo* colonization, Niu uses nickel chelated to a nickelophore (as reported for the NikABC transporter), and that this complexed form is not efficiently recognized by NixA. Thus, *H*. *pylori* has two versatile systems that are required to allow efficient and persistent colonization of a hostile environment.

In conclusion, we have identified a novel *H*. *pylori* nickel transporter that is essential for colonization and together with NixA represent the two sole nickel transporters in *H*. *pylori*. These transporters have been acquired by gastric *Helicobacter* species through horizontal transfer. Together with the acquisition of the Ni(II)-binding proteins, Hpn and Hpn-2, this further highlights how important it has been during evolution of gastric *Helicobacter* species to increase their capacity to accumulate intracellular nickel without toxicity and thereby optimize urease activity required for persistent colonization of the acidic stomach.

## Materials and Methods

### Ethics statement

Experiments in mice were carried out in strict accordance with the recommendations in the Specific Guide for the Care and the Use of Laboratory Animals of the Institut Pasteur, according to the European Directive (2010/63/UE) and the corresponding French law on animal experimentation (Arrêtés de 1988). The protocol has been approved by the Committee of Central Animal Facility Board of the Institut Pasteur. To follow the new European directives, the project was approved by the CETEA, Comité d’éthique en Expérimentation Animale of the Institut Pasteur (*#*2013–0051) and by the Ministère de l’Enseignement Supérieur et de la recherche (#751501).

### Bacterial strains and growth conditions

All plasmid constructions of this work were made in the *E*. *coli* strain BTH101 [68] grown on solid or liquid Luria-Bertani medium [69] supplemented with spectinomycin 100 μg.mL^-1^, chloramphenicol 30 μg.mL^-1^, ampicillin 100 μg.mL^-1^ or kanamycin 40 μg.mL^-1^, when required.

The *H*. *pylori* strains used in this study (S1 Table) are G27 [50], B128 [70,71], B128-S (this study), SS1 [72] and 26695 [73] *H*. *pylori* strains were grown on Blood Agar Base 2 (Oxoid) plates supplemented with 10% defibrinated horse blood and with the following antibiotics-antifungal cocktail: amphotericin B 2.5 μg.mL^-1^, polymyxin B 0.31 μg.mL^-1^, trimethoprim 6.25 μg.mL^-1^ and vancomycin 12.5 μg.mL^-1^. For liquid cultures, we used Brucella broth, designated BB (BD Difco) supplemented with 10% fetal calf serum (FCS, Eurobio) or with 0.2% ß-cyclodextrin (Sigma) (designated here BBß), with the antibiotics-antifungal cocktail and the selective antibiotic when required. Selection and growth of *H*. *pylori* mutants and transformants were performed using kanamycin 20 μg.mL^-1^, chloramphenicol 6 μg.mL^-1^ or streptomycin 10 μg.mL^-1^.

### Molecular techniques

Molecular biology experiments were performed according to standard procedures [74] and the supplier (Fermentas) recommendations. NucleoBond Xtra Midi Kit (Macherey-Nagel) and QIAamp DNA Mini Kit (Qiagen) were used for plasmid preparations and *H*. *pylori* genomic DNA extractions, respectively. PCR were performed either with Taq Core DNA polymerase (MP Biomedicals), or with Phusion Hot Start DNA polymerase (Finnzymes) when the product required high fidelity polymerase. The pGEMT vector (Promega) was used to construct in *E*. *coli* the suicide plasmids that served for mutagenesis in *H*. *pylori*.

### Construction of unmarked *H. pylori* mutants

Unmarked *niuD* and *niuB* deletion mutants of *H*. *pylori* were constructed by allelic exchange as described [53] using *H*. *pylori* suicide plasmids derived from pGEMT, in which about 500 bp of the 5’-end and the 3’-end regions immediately flanking the open reading frame of the target gene were cloned using PCR fragments generated with the primers indicated in S2 Table on each side of a difH-cat-rpsL-difH cassette amplified with primers difHrpsLcat-1 and difHrpsLcat-2. The *H*. *pylori* mutants were obtained by natural transformation of B128-S, a streptomycin resistant derivative of B128, with 1 μg of a preparation of the suicide plasmid DNA and selection on blood agar plates containing chroramphenicol 6 μg.mL^-1^ (for G27 and B128-S) or 10 μg.mL^-1^ (for SS1) as described previously [75]. Removal of the cassette was achieved by plating the Cm^R^ clones on blood agar plates containing streptomycin 10 μg. mL^-1^. Deletion of the gene of interest was verified by PCR and sequencing of the gene region.

### Construction of plasmids and complemented mutants

The mutants were complemented through two strategies. One was by introducing the wild type copy of the gene to be complemented under the control of the *ureI* promoter on the chromosome, at a neutral site that was shown to affect neither *in vitro* growth nor mouse colonization as described in [22,54]. The corresponding constructs were designated “c-X” for chromosomally inserted, X being the name of the gene. The second one was by cloning the gene under the control of an IPTG inducible promoter in the pILL2157 *E*. *coli*/*H*. *pylori* shuttle vector [76], the corresponding constructs were designated “p-X” for inserted on a plasmid, X being the name of the gene. For the chromosomal complementation, the *niuDE*, *niuB1 and niuB2* genes were PCR-amplified from the B128 chromosome and cloned into the *Eco*RV sites of pIRC plasmid in which we had introduced the P_*ureI*_ promoter [22] (PCR using the couple of primer IR-UP/IR-DO), allowing the transcription of the genes under the control of P_*ureI*_ promoter. The pIRC(P_*ureI*_) derivatives were used to transform strain 26695 selecting for chloramphenicol resistance. We verified the presence of the wild type copy of the genes at the correct location by sequencing PCR amplification products made with primers hp203/hp204. The chromosomal DNAs from correct chloramphenicol resistant clones (*i*.*e*. where the wild type genes and the *cat* cassette integrated by homologous recombination between genes *hp0203* and *hp0204*) were then used to introduce the *niuD*, *niuB1 or niuB2* genes into various strains by natural transformation and selection for chloramphenicol resistance. For cloning into pILL2157, the genes were PCR-amplified from the B128 chromosome using the couple of primers pil-UP/pil-DO and cloned into the *Nde*I/*Eco*RV sites (for *niuD* and *niuDE*) or the *Nd*eI/*Bam*HI sites (for *niuB1* and *niuB2*) of pILL2157. To construct the P_*fecA3*_::*lacZ* reporter, we PCR-amplified the promoter sequence from the B128 chromosome using the couple of primers PfecA3-UP/ PfecA3-DO and cloned it into the *Bgl*II/*Spe*I sites of pILL2157. ß-galactosidase activities of strains carrying this fusion were measured with the classical *E*. *coli* protocol [69] adapted to *H*. *pylori* as described in [22].

### Mouse model of colonization

NMRI-specific pathogen-free mice (Charles River Laboratories) were orogastrically inoculated with 10^9^ CFU of *H*. *pylori* strains prepared in 100 μL of peptone broth. One month after inoculation, mice were sacrificed and stomachs were crushed in peptone broth (as described in [58]). Viable *H*. *pylori* colonizing the stomach were enumerated by culture of serial dilutions of homogenized tissue on blood agar plates containing in addition bacitracin (200 μg.mL^-1^) and nalidixic acid (10 μg.mL^-1^).

### Measurement of metal sensitivity

To test metal sensitivity during growth in liquid, *H*. *pylori* cells were inoculated at OD_600_ 0.05 in 10 mL liquid Brucella-Broth containing 10% FCS either without or with 0.5, 1.5 or 2 mM NiCl_2_, with 1 or 2 mM for ZnCl_2_, CuCl_2_, MnSO_4_, with 0.01 mM CoCl_2_ and 0.1 mM of a colloidal preparation of Bismuth subcitrate potassium. Bacterial growth was monitored 24 hours later by measuring OD_600_. The data correspond to at least three independent experiments with two replicates per experiment.

### Nickel content measurements by Inductively Coupled Plasma Optical Emission Spectrometry (ICP-OES)

Overnight liquid cultures of *H*. *pylori* strain were grown until OD_600_ 0.9 at 37°C in 6 ml Brucella-Broth with FCS, then 10 μM or 100 μM NiCl_2_ were added and the cultures were left to grow until OD_600_ 6. Then, the 6 ml of culture were centrifuged at 4000 g at 4°C for 25 min through 400 μL of a 1:2 mixture of the silicone oils AR20/AR200 (Wacker) in order to separate the cells from the medium. Cells were lysed with 400 μL 0.2 M NaOH/1% SDS for 60 min at 95°C. Samples were calibrated by protein concentration measurements with the DC Protein Assay kit (BioRad). Then, the samples were mineralized overnight at 80°C with 300 μL of ultrapure 70% nitric acid (JT Baker) and diluted to 1/20 in ultrapure water. Nickel contents were measured by ICP-OES using a Vista MPX spectrometer (Varian). The content of Ni(II) was determined using a curve established with certified ICP grade standards. The measurement of each strain in each condition was performed in triplicates in at least two independent experiments.

### Transport of radiolabelled nickel

The procedure was adapted from our previously published protocol [26]. As shown in that paper, this test is only functioning at pH 5, presumably because the outer membrane transporter FrpB4 is acid-activated [26]. Overnight 10 mL precultures of *H*. *pylori* B128-S wild-type and mutant cells were diluted to OD_600_ 0.25 into 10 mL fresh BB medium supplemented with fetal calf serum and incubated in microaerophily with shaking at 37°C. When the cultures reached OD_600_ 0.5, cells were harvested, washed twice in fresh BB medium with β-cyclodextrin (BBβ), resuspended in the same volume of BBβ, that was adjusted to pH 5, and shaken during 20 minutes in microaerophily, at 37°C. Radiolabelled ^63^NiCl_2_ (3.71 mCi/mL, 16.3 mCi/mg), was isotopically diluted 10-fold with cold NiCl_2_ and added to each culture to a final concentration of 10 μM. ^63^NiCl_2_ was supplied by the United States Department of Energy Office of Science by the Isotope Program in the Office of Nuclear Physics. Kinetics were performed during 45 minutes with a time point before nickel addition, and time points at 5, 20, 30 and 45 minutes after nickel addition. Aliquots of 1 mL were withdrawn, immediately vacuum filtered through 0.45 μm pore-size cm filters (∅ = 2.5; Millipore) and washed with 10 mL of 50 mM Tris-HCl (pH 7.0) containing 1 mM cold NiCl_2_ to avoid unspecific binding. Two series of experiments were performed and each time point was measured in duplicates. Filters were dried and radioactivity was counted. Controls comprised counting of the filters after incubation in the absence of bacteria or with lysed cells and background values were subtracted. Uptake rates were calculated as cpm of accumulated ^63^Ni as a function of time.

### Urease activity measurements and acid resistance assays

Urease activity of whole *H*. *pylori* cells was assayed by measuring ammonia production using the Ammonia-Assay Kit (Sigma) as described before [77]. *H*. *pylori* bacteria grown on blood agar plates for 24 hours were inoculated at an OD_600_ 0.05 in BB liquid medium with fetal calf serum (10% v/v) and grown overnight. This preculture was used to inoculate the bacteria at an OD_600_ 0.25 in liquid BB with 10% (v/v) fetal calf serum without added nickel. When an OD_600_ of 0.5 was reached, cells were washed once in phosphate buffer saline (Sigma) and resuspended to the same OD_600_ in BBβ pH 5 or pH 7 and incubated 1 hour at 37°C under microaerophily with shaking. From this culture, log-phase bacteria (0.5 OD_600_) were harvested, washed once with phosphate-buffered saline and resuspended in 1 ml of buffer (citric acid, 0.1 M; Na_2_HPO_4_, 0.2 M at pH 5) containing 6 mM urea. The data correspond to at least three independent experiments with two technical replicates each time. Aliquots were taken after 8 min of incubation at 37°C and centrifuged to pellet the bacteria. The NH_3_ concentration in the supernatant was measured with the ammonia-assay kit according to the manufacturer’s (SIGMA) instructions. One unit (U) of urease activity was defined as the amount of enzyme that generates 1 μmol ammonia per min per mg of total proteins. For clarity, results were represented in the graphs after normalizing absolute values relative to the wild type strain.

For acid resistance assays, *H*. *pylori* bacteria were grown as described for urease assays. When an OD_600_ of 0.5 was reached, cells were washed once in phosphate buffer saline (Sigma) and resuspended to the same OD_600_ in BBβ pH 5 and incubated 1 hour at 37°C under microaerophily with shaking. From this culture, log-phase bacteria (0.5 OD_600_) were harvested and washed once with phosphate-buffered saline and resuspended in phosphate-buffered saline pH 7 (control) or pH 2 (assays). Cells exposed to acidic conditions (pH 2) were supplemented or not with 6 mM urea. After 40 min exposure, an aliquot of 0.2 mL of each bacterial suspension was centrifuged (3 min, 8000 x *g*), and 10 μL of supernatant was applied onto pH-paper. Ten μL aliquots of standard solutions of known pH were used to produce a control pH scale (pH 2, 4, 5, 7 and 10). Aliquots of 20 μL of each strain was taken in parallel and added into individual wells of a 96-well plate each containing 80 μL of BB medium and 25 μL of Alamar Blue reagent to test for viability (Thermo Scientific) and grown at 37°C under microaerophily until staining developed (typically 1 to 2 hours). The Alamar Blue test uses the cell-permeable resazurin blue molecule and measures the capacity of viable cells to keep an intracellular reducing environment where resazurin is converted to a red resorufin derivative [59].

### Statistical analysis

The Student’s t test was used to determine significance of the means of the data. The Mann-Whitney test was used for mouse colonization assay to compare colonization loads.

### Genomic analyses

To determine the distribution of nickel transporter proteins, hydrogenase and urease among *Helicobacter* species, we used MycoHIT as previously described [60,61]. Briefly, we performed an alignment search with the StandAlone TBLASTN program [78], using the proteins sequences of HydA, UreB, UreI, NixA, NiuB, NiuD, NiuE from *H*. *pylori* B128, NikA, NikB, NikCD, NikE from *H*. *hepaticus* ATCC51478, NikZ from *H*. *felis* ATCC 49179 and OppB-, OppC-, OppD- and OppE-like proteins from *H*. *felis* ATCC 49179, genes that are situated downstream of NikZ (operon *nikZ-oppBCDE*) as the query sequences to search for matches in the genomic DNA of other organisms. To categorically assign that there was no hit, we employed the default E-value (or Expectation value) of e-10 which is the default value provided at NCBI and has been used in a similar study [60,61]. Thus, if the statistical significance ascribed to a comparison is greater than this E value, we assigned a percentage of similarity and identity of 0% to that comparison. As a database, we used the genomes of *H*. *acinonychis* str. Sheeba (NC_008229.1), *H*. *bilis* ATCC 43879 (NZ_KI392032.1), *H*. *canadensis* MIT 98–5491 (NZ_CM000776.2), *H*. *canis* NCTC 12740 (NZ_KI669458.1), *H*. *cetorum* MIT 00–7128 (NC_017737.1), *H*. *felis* ATCC 49179 (NC_014810.2), *H*. *fennelliae* MRY12-0050 (NZ_BASD00000000.1), *H*. *hepaticus* ATCC 51449 (NC_004917.1), *H*. *macacae* MIT 99–5501 (NZ_AZJI00000000.1), *H*. *mustelae* 12198 (NC_013949.1), *H*. *pametensis* ATCC 51478 (NZ_JADE00000000.1), *H*. *pullorum* MIT 98–5489 (NZ_ABQU00000000.1), *H*. *rodentium* ATCC 700285 (NZ_JHWC00000000.1), *H*. *suis* HS1 (NZ_ADGY00000000.1), *H*. *winghamensis* ATCC BAA-430 (NZ_ACDO00000000.1), *H*. *bizzozeronii* CIII-1 (NC_015674.1), *H*. *pylori* strain 26695 (NC_000915.1), *H*. *cetorum* MIT 99–5656 (NC_017735.1), *H*. *heilmannii* ASB1.4 (NC_019674.1), *H*. *cinaedi* ATCC BAA-847 (NC_020555.1), *H*. *apodemus* strain MIT-03-7007 (NZ_JRPC00000000.1), *H*. *muridarum* strain ST1 (NZ_JRPD00000000.1), *H*. *trogontum* strain ATCC 700114 (NZ_JRPL00000000.1), *H*. *ailurogastricus* (NZ_CDMG00000000.1).

### Phylogenetic analyses

We retrieved sequences of NixA (HPB8_1196), NiuB1 (HPB8_1657), and NiuD (HPB8_663) from *H*. *pylori* B128 that were annotated in the KEGG database (http://www.genome.jp/kegg/). These were used as queries to search for homologs in complete proteomes (non redundant protein database) at the National Center for Biotechnology Information (NCBI, http://www.ncbi.nlm.nih.gov/) using the BlastP program [78] (default parameters). For each protein, homologs with amino acid identity >35% were extracted. Protein sequences were aligned with the Muscle program [79] and the resulting multiple alignments were trimmed with the BMGE software [80] with a BLOSUM30 substitution matrix. Maximum likelihood (ML) trees of NixA, NiuB and NiuD were then reconstructed using IQ-TREE [81] and the LG+Г4 model. The branch robustness of the ML trees was estimated with the nonparametric bootstrap procedure (100 replicates of the original datasets). Trees were drawn and annotated with the FigTree (v1.4.2) program (http://www.webcitation.org/getfile?fileid=27177ee8dd2f34cfd254b9c5e6c6fdf4b65329f6).

## Supporting Information

S1 Fig NiuD and NixA mediate *H*. *pylori* sensitivity to high nickel concentrations in a B128-S *Δhpn* context. Effect of 0.5 and 1 mM NiCl_2_ on growth of *H*. *pylori* B128-S *Δhpn* parental strain and isogenic mutants. The results are presented as % of growth in the presence of nickel relative to growth without nickel after 24h incubation. The data correspond to the mean value of three independent experiments and error bars represent the standard deviation. *** indicates that the mean value is significantly different from that of the wild type strain (*P* ≤ 0.001).
(TIF)

S2 Fig NiuD and NixA control the intracellular nickel content in *H*. *pylori* in a wild type and B128-S *Δhpn* context. Nickel amounts measured by Inductively Coupled Plasma Optical Emission Spectrometry (ICP-OES) and expressed as μg of nickel. g^-1^ of proteins. Strains were grown either without added nickel, with 10 μM or with 100 μM nickel. The data correspond to the mean value of three independent experiments and error bars represent the standard deviation. *** indicates that the mean value is significantly different from that of the wild type strain (*P* ≤ 0.001).
(TIF)

S3 Fig Distribution of NixA, NiuB, NiuD, NiuE, UreI (urea channel) and subunits of urease (UreB), hydrogenase (HydA), NikABCDE and NikZOppBCDE throughout the *Helicobacter* genus. Protein homologs of each query protein were detected with the MycoHIT program and the table represents the resulting hits with details on sequence identities. The lower panel presents examples of the organization of genes encoding NiuB, NiuD, NiuE, NixA (*H*. *pylori* 26695, *H*. *cetorum* MIT 00–7128, *H*. *mustelae*), NikABCDE (*H*. *hepaticus*), NikZOppBCDE (*H*. *felis*, *H*. *suis*). NixA is present only in gastric species, while *niuBDE* is found as a putative operon (in *H*. *fennelliae*, an EH species), or as an *niuDE* operon separated from *niuB* gene(s) (*H*. *pylori*, *H*. *cetorum*, *H*. *acinonychis*, *H*. *mustelae*). NikZ, a periplasmic nickel binding protein, is always encoded by a gene lying within a putative 5-genes operon, annotated *nikZoppBCDE*, where *oppBCDE* encodes components of an ABC transport system (*oppBC* encoding permease components and *oppDE* ATPase subunits). Interestingly, in addition to this ABC transport system, *H*. *felis* possesses two *nixA* genes. The *nikZoppBCDE* operon is found in all *heilmannii*-like species (*H*. *suis*, *H*. *heilmannii*, *H*. *bizzozeronni*, *H*. *felis* and *H*. *ailurogastricus*). In *H*. *hepaticus*, the *nikABCDE* operon encoding a nickel-specific ABC transporter is found downstream the *ureAB-ureEFGH* cluster encoding urease subunits UreA and UreB and accessory proteins necessary for urease maturation (UreE, F, G and H). This *nikABCDE* operon is found in several EH species.
(TIF)

S4 Fig Maximum likelihood (ML) phylogeny of NixA. The tree was inferred with IQ-TREE and the LG+Г4 model and rooted with sequences of Clostridia. It contains the 86 sequences closest to the NixA of *H*. *pylori*. Values at nodes represent statistical confidence based on 100 bootstrap replicates of the original dataset. The scale bar represents the average number of substitutions per site. Prokaryotic lineages are indicated on the right side. Important nodes are colored in red.
(TIF)

S5 Fig Maximum likelihood (ML) phylogeny of NiuB (unrooted). The tree was inferred with IQ-TREE and the LG+Г4 model. Values at nodes represent statistical confidence based on 100 bootstrap replicates of the original dataset. The scale bar represents the average number of substitutions per site. Colors correspond to various prokaryotic lineages. Note that NiuB sequences from epsilon-proteobacteria (blue background) are scattered and that NiuB from gastric *Helicobacter* are clustered in a single clade with NiuB from *C*. *sputorum* and *H*. *fennelliae* (all epsilonproteobacteria, light pink background).
(TIF)

S6 Fig Maximum likelihood (ML) phylogeny of NiuD (unrooted). The tree was inferred with IQ-TREE and the LG+Г4 model. Values at nodes represent statistical confidence based on 100 bootstrap replicates of the original dataset. The scale bar represents the average number of substitutions per site. Colors correspond to various prokaryotic lineages. NiuD sequences from epsilon-proteobacteria (in blue) are also scattered. NiuB from gastric *Helicobacter* are, again, clustered in a single clade with NiuB from *C*. *sputorum* and *H*. *fennelliae* (pink background).
(TIF)

S7 Fig Maximum likelihood (ML) phylogeny of NiuB and NiuD (close-in views from S5 and S6). Colors correspond to various prokaryotic lineages that are indicated on the right side. Gastric *Helicobacter* species are highlighted with a light grey background. For each species, genetic organization of the *niuB* gene is indicated (associated or separated from *niuD* and *niuE*) in the upper tree, and information on the organization of *niuD* and *niuE* genes are indicated in the bottom tree. Cartoons codes are indicated at the bottom. Genes associated within a putative operon are highlighted with a light green background.
(TIF)

S8 Fig Structural analysis of NiuB. *Upper panel*–Multiple alignment of NiuB (CeuE) from *H*. *pylori* strain G27, NiuB1 and NiuB2 from strain B128. The putative signal peptide of the three proteins (predicted with SignalP) is indicated with their putative cleavage site. Alignments show that NiuB1 is closely related to NiuB (G27), while NiuB2 shows several differences, such as insertions and substitutions. This pattern is representative of the differences between the NiuB/NiuB1 proteins and NiuB2 proteins. Blue boxes indicate residues involved in Ni(II)-(L-His)_2_ binding and blue stars highlight the two key residues specifically involved in metal binding. Red boxes and red arrows indicate residues close to the binding site that are different between NiuB/NiuB1 and NiuB2. Black boxes emphasize the two residues (Glu110 and Glu236) that have been proposed to be involved in binding onto the NiuD permease. Green arrows show the differences between the two types of proteins. *Middle panel–*Representation of the 3D structure of NiuB (G27) (PDB code: 4LS3, [56]) with the ligand represented as solid spheres. Residues indicated by green arrows in the upper panel are represented here with green surfaces and show that they all lie on the surface of NiuB. Glu110 and Glu236 residues are conserved in both NiuB1 and NiuB2, indicating that they could bind NiuD similarly. *Lower panel–*Close-in view of the binding site of NiuB (G27, [56]). Residues involved in direct nickel binding are indicated with a blue star and several other conserved residues are indicated in black. Residues from the binding site that change between NiuB/NiuB1 (black) and NiuB2 (green) are indicated. The binding pocket is slightly different between both types of NiuB, with Val104, Ile208 and Asp209 in NiuB/NiuB1 being replaced by Thr106, Ile208 and Ser211 in NiuB2. These differences are putatively accounting for the differences observed in nickel uptake efficiencies between NiuB1 and NiuB2 in our experiments.
(TIF)

S1 Table Strains and plasmids used in this study. (DOCX)

S2 Table Oligonucleotides used in this study. (DOCX)
