# Supplementary figures and images for: Characterization in Helicobacter pylori of a Nickel Transporter Essential for Colonization That Was Acquired during Evolution by Gastric Helicobacter Species
Source: PLoS Pathog. 2016 Dec 6;12(12):e1006018. doi: 10.1371/journal.ppat.1006018 (PMC5140060; doi:10.1371/journal.ppat.1006018)

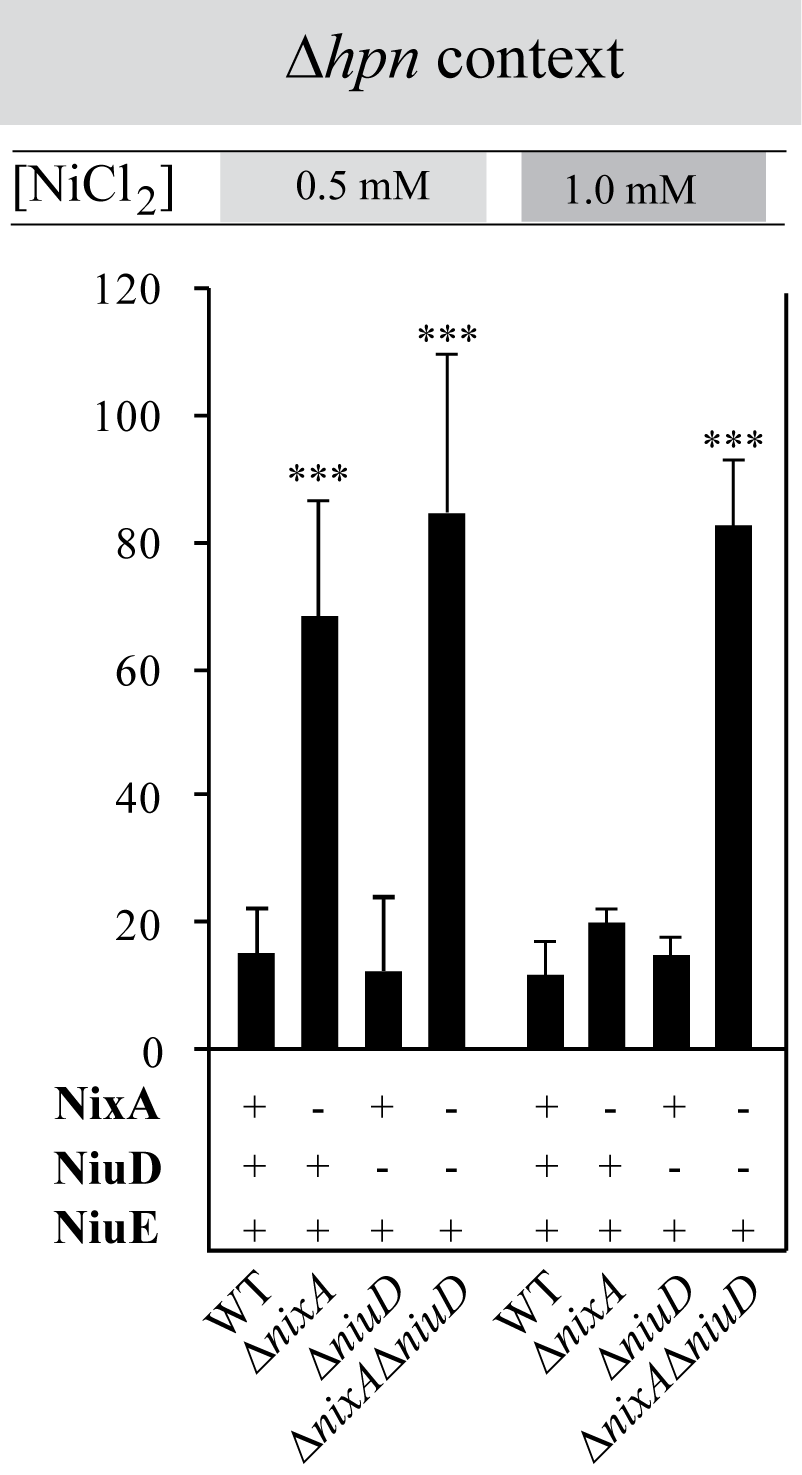

Supplement: S1 Fig — Effect of 0.5 and 1 mM NiCl2 on growth of H. pylori B128-S Δhpn parental strain and isogenic mutants. The results are presented as % of growth in the presence of nickel relative to growth without nickel after 24h incubation. The data correspond to the mean value of three independent experiments and error bars represent the standard deviation. *** indicates that the mean value is significantly different from that of the wild type strain (P ≤ 0.001). (TIF) [file ppat.1006018.s001.tif]

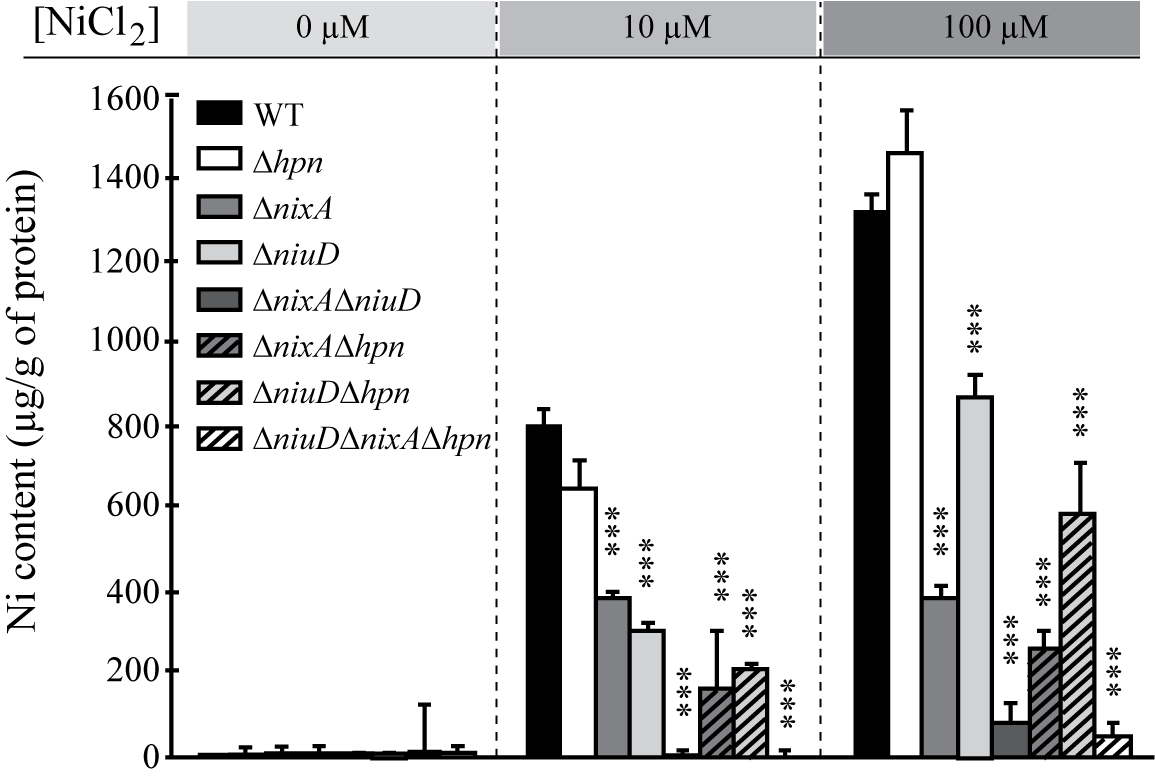

Supplement: S2 Fig — Nickel amounts measured by Inductively Coupled Plasma Optical Emission Spectrometry (ICP-OES) and expressed as μg of nickel. g-1 of proteins. Strains were grown either without added nickel, with 10 μM or with 100 μM nickel. The data correspond to the mean value of three independent experiments and error bars represent the standard deviation. *** indicates that the mean value is significantly different from that of the wild type strain (P ≤ 0.001). (TIF) [file ppat.1006018.s002.tif]

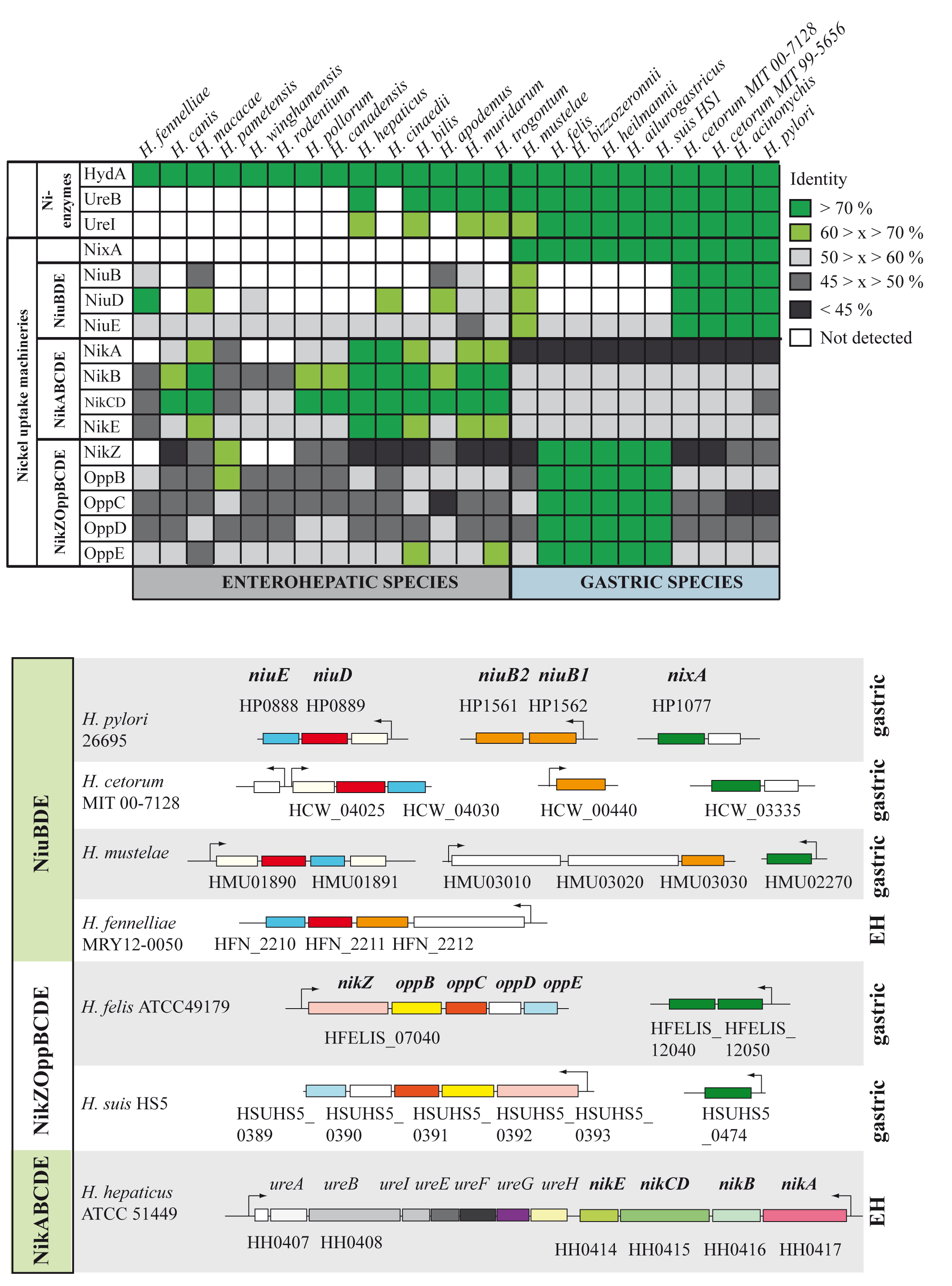

Supplement: S3 Fig — Protein homologs of each query protein were detected with the MycoHIT program and the table represents the resulting hits with details on sequence identities. The lower panel presents examples of the organization of genes encoding NiuB, NiuD, NiuE, NixA (H. pylori 26695, H. cetorum MIT 00–7128, H. mustelae), NikABCDE (H. hepaticus), NikZOppBCDE (H. felis, H. suis). NixA is present only in gastric species, while niuBDE is found as a putative operon (in H. fennelliae, an EH species), or as an niuDE operon separated from niuB gene(s) (H. pylori, H. cetorum, H. acinonychis, H. mustelae). NikZ, a periplasmic nickel binding protein, is always encoded by a gene lying within a putative 5-genes operon, annotated nikZoppBCDE, where oppBCDE encodes components of an ABC transport system (oppBC encoding permease components and oppDE ATPase subunits). Interestingly, in addition to this ABC transport system, H. felis possesses two nixA genes. The nikZoppBCDE operon is found in all heilmannii-like species (H. suis, H. heilmannii, H. bizzozeronni, H. felis and H. ailurogastricus). In H. hepaticus, the nikABCDE operon encoding a nickel-specific ABC transporter is found downstream the ureAB-ureEFGH cluster encoding urease subunits UreA and UreB and accessory proteins necessary for urease maturation (UreE, F, G and H). This nikABCDE operon is found in several EH species. (TIF) [file ppat.1006018.s003.tif]

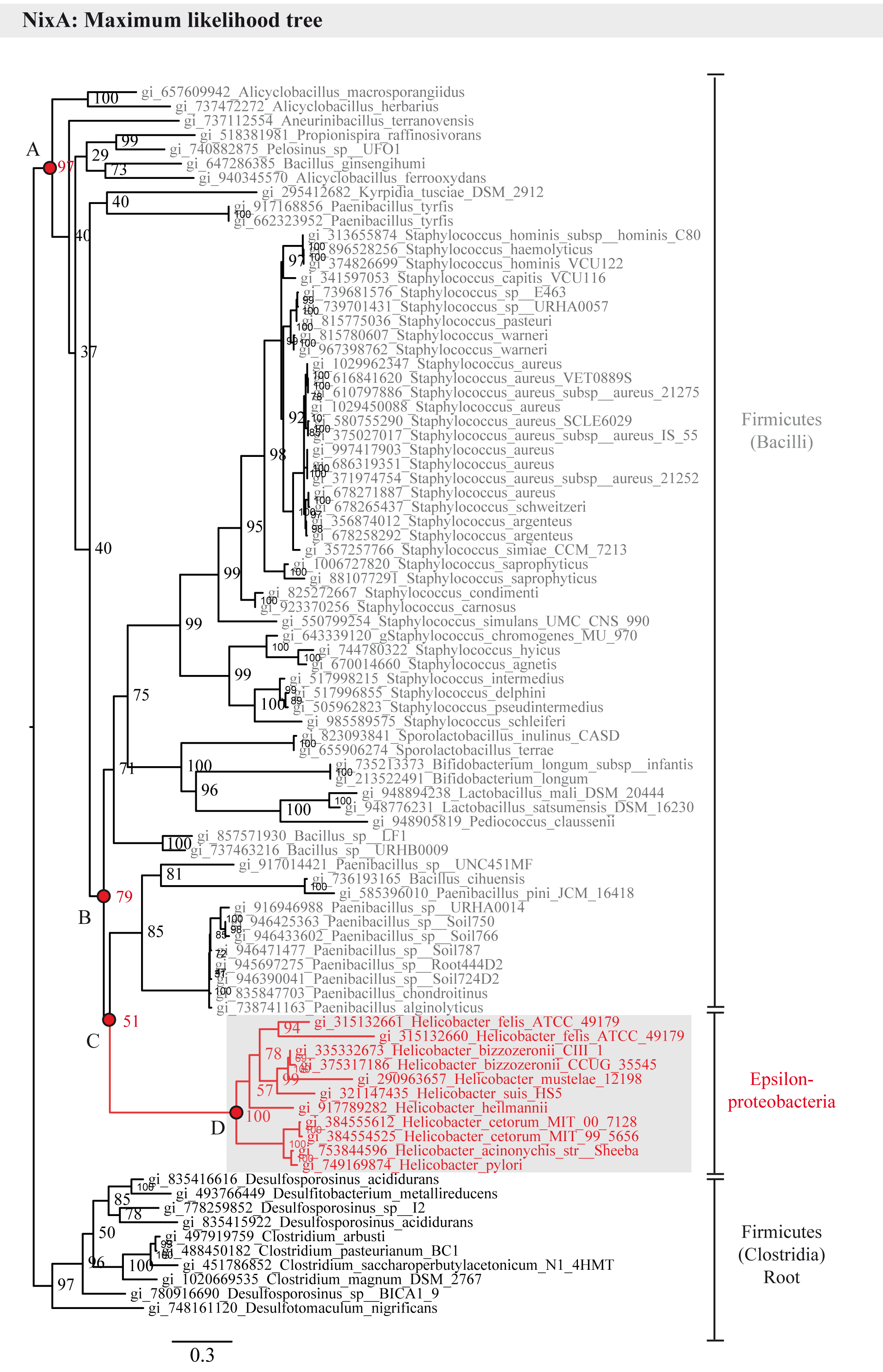

Supplement: S4 Fig — The tree was inferred with IQ-TREE and the LG+Г4 model and rooted with sequences of Clostridia. It contains the 86 sequences closest to the NixA of H. pylori. Values at nodes represent statistical confidence based on 100 bootstrap replicates of the original dataset. The scale bar represents the average number of substitutions per site. Prokaryotic lineages are indicated on the right side. Important nodes are colored in red. (TIF) [file ppat.1006018.s004.tif]

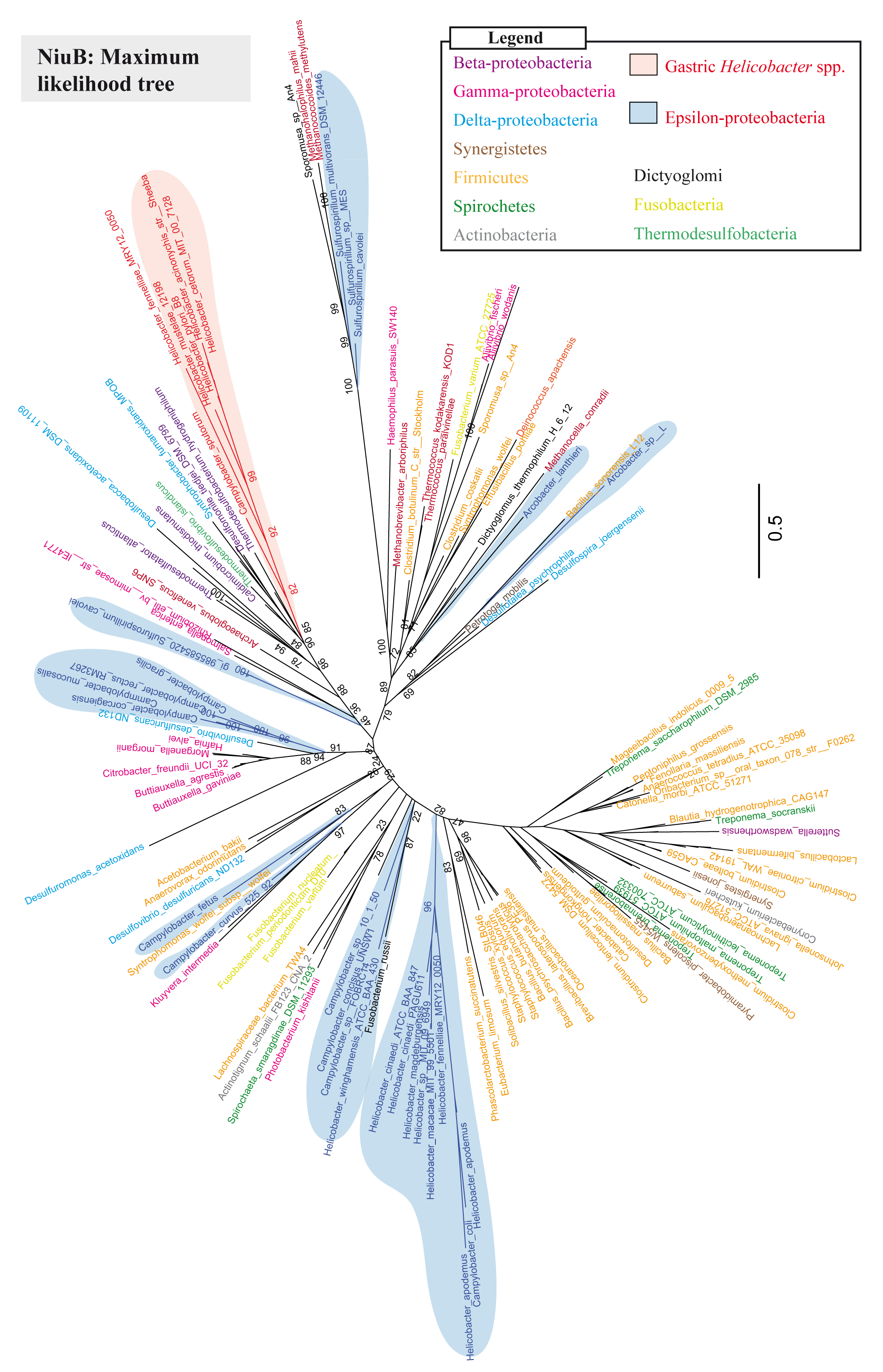

Supplement: S5 Fig — The tree was inferred with IQ-TREE and the LG+Г4 model. Values at nodes represent statistical confidence based on 100 bootstrap replicates of the original dataset. The scale bar represents the average number of substitutions per site. Colors correspond to various prokaryotic lineages. Note that NiuB sequences from epsilon-proteobacteria (blue background) are scattered and that NiuB from gastric Helicobacter are clustered in a single clade with NiuB from C. sputorum and H. fennelliae (all epsilonproteobacteria, light pink background). (TIF) [file ppat.1006018.s005.tif]

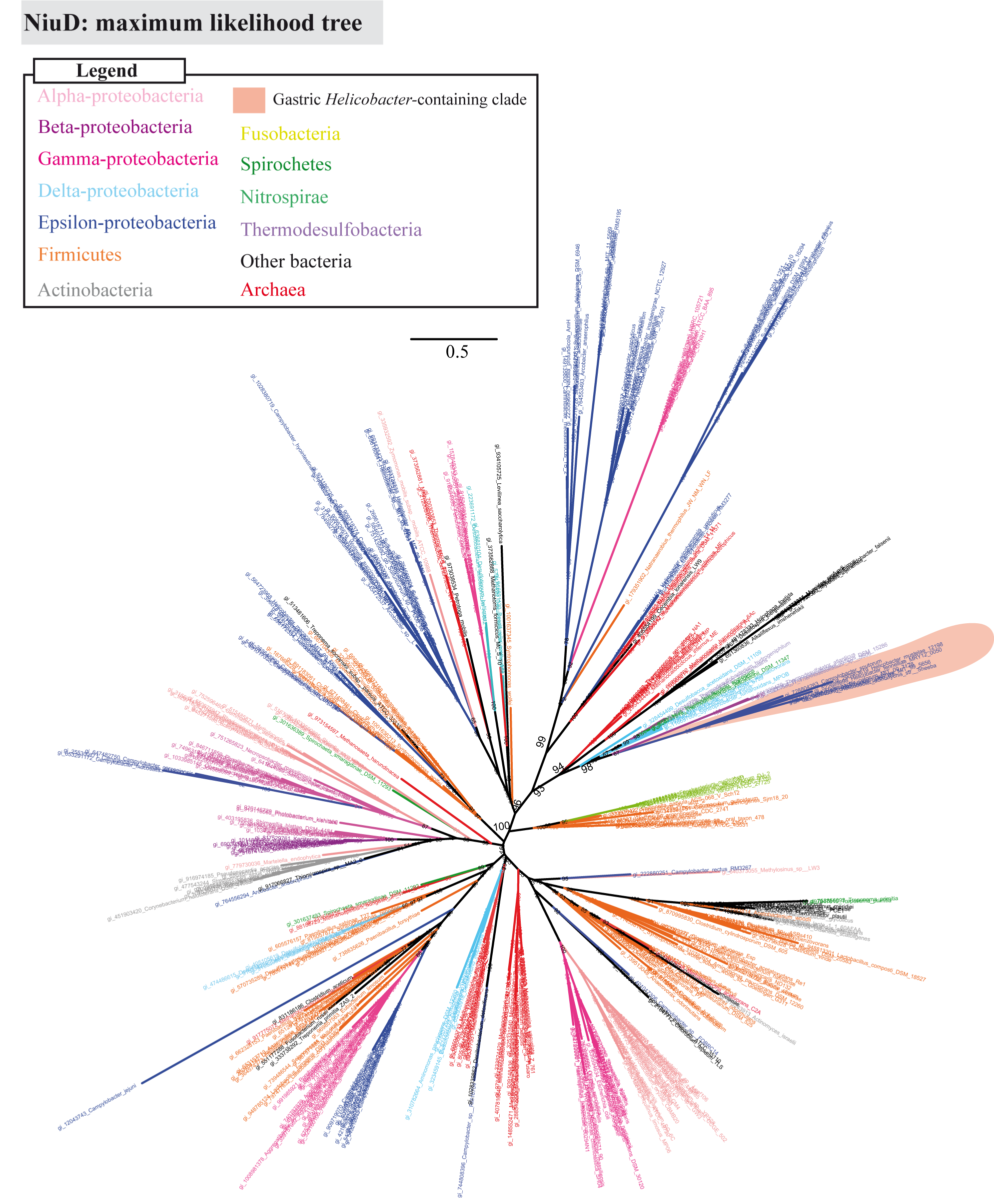

Supplement: S6 Fig — The tree was inferred with IQ-TREE and the LG+Г4 model. Values at nodes represent statistical confidence based on 100 bootstrap replicates of the original dataset. The scale bar represents the average number of substitutions per site. Colors correspond to various prokaryotic lineages. NiuD sequences from epsilon-proteobacteria (in blue) are also scattered. NiuB from gastric Helicobacter are, again, clustered in a single clade with NiuB from C. sputorum and H. fennelliae (pink background). (TIF) [file ppat.1006018.s006.tif]

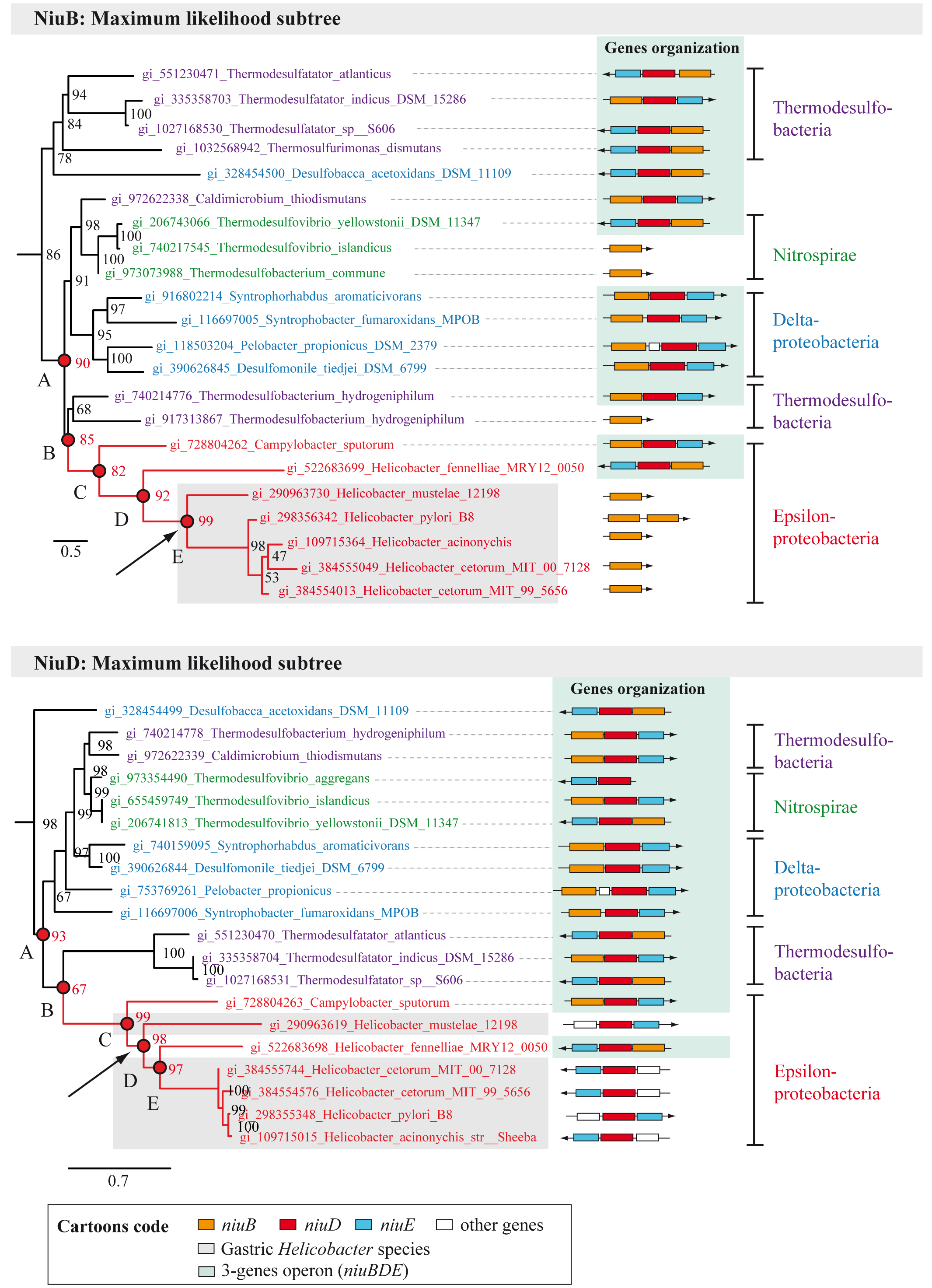

Supplement: S7 Fig — Colors correspond to various prokaryotic lineages that are indicated on the right side. Gastric Helicobacter species are highlighted with a light grey background. For each species, genetic organization of the niuB gene is indicated (associated or separated from niuD and niuE) in the upper tree, and information on the organization of niuD and niuE genes are indicated in the bottom tree. Cartoons codes are indicated at the bottom. Genes associated within a putative operon are highlighted with a light green background. (TIF) [file ppat.1006018.s007.tif]

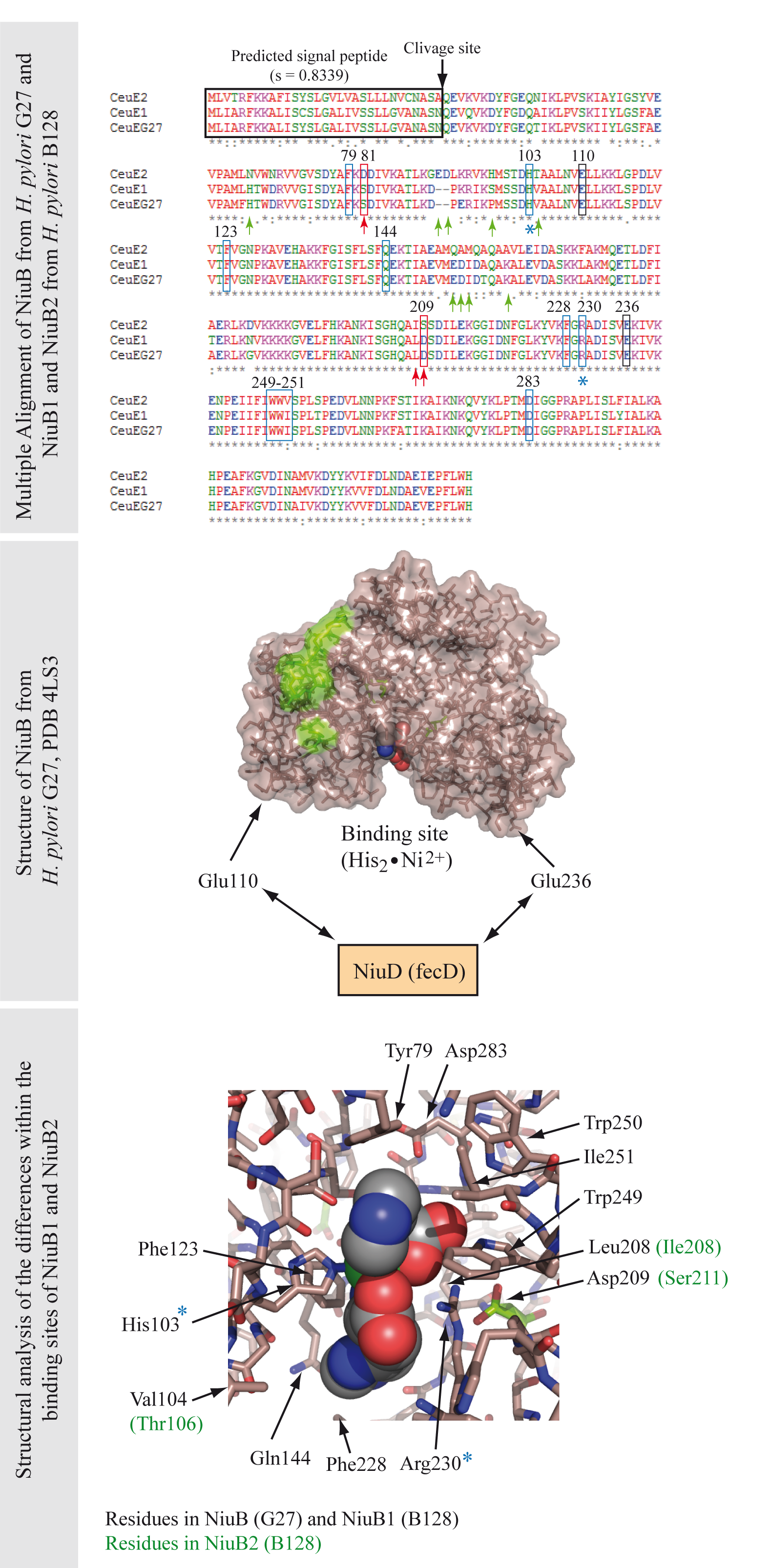

Supplement: S8 Fig — Upper panel–Multiple alignment of NiuB (CeuE) from H. pylori strain G27, NiuB1 and NiuB2 from strain B128. The putative signal peptide of the three proteins (predicted with SignalP) is indicated with their putative cleavage site. Alignments show that NiuB1 is closely related to NiuB (G27), while NiuB2 shows several differences, such as insertions and substitutions. This pattern is representative of the differences between the NiuB/NiuB1 proteins and NiuB2 proteins. Blue boxes indicate residues involved in Ni(II)-(L-His)2 binding and blue stars highlight the two key residues specifically involved in metal binding. Red boxes and red arrows indicate residues close to the binding site that are different between NiuB/NiuB1 and NiuB2. Black boxes emphasize the two residues (Glu110 and Glu236) that have been proposed to be involved in binding onto the NiuD permease. Green arrows show the differences between the two types of proteins. Middle panel–Representation of the 3D structure of NiuB (G27) (PDB code: 4LS3, [56]) with the ligand represented as solid spheres. Residues indicated by green arrows in the upper panel are represented here with green surfaces and show that they all lie on the surface of NiuB. Glu110 and Glu236 residues are conserved in both NiuB1 and NiuB2, indicating that they could bind NiuD similarly. Lower panel–Close-in view of the binding site of NiuB (G27, [56]). Residues involved in direct nickel binding are indicated with a blue star and several other conserved residues are indicated in black. Residues from the binding site that change between NiuB/NiuB1 (black) and NiuB2 (green) are indicated. The binding pocket is slightly different between both types of NiuB, with Val104, Ile208 and Asp209 in NiuB/NiuB1 being replaced by Thr106, Ile208 and Ser211 in NiuB2. These differences are putatively accounting for the differences observed in nickel uptake efficiencies between NiuB1 and NiuB2 in our experiments. (TIF) [file ppat.1006018.s008.tif]
