# Supplementary material for: Characterization in Helicobacter pylori of a Nickel Transporter Essential for Colonization That Was Acquired during Evolution by Gastric Helicobacter Species
Source: PLoS Pathog. 2016 Dec 6;12(12):e1006018. doi: 10.1371/journal.ppat.1006018 (PMC5140060; doi:10.1371/journal.ppat.1006018)
Supplement: S1 Table — (DOCX) [file ppat.1006018.s009.docx]

**Supplementary S1 Table: strains and plasmids used in this study**

| **Strains** | **Relevant characteristics** | **Reference** |
| --- | --- | --- |
| *Escherichia coli* |  |  |
| BTH101 | *F^-^, cya-99, araD139, galE15, galK16, rpsL1 (Str^R^), hsdR2, mcrA1, mcrB1* | [68] |
| *Helicobacter pylori* |  |  |
| B128 | Sequenced parental strain | [70,71] |
| B128-S | B128 streptomycin resistance (point mutation) | This work |
| B128-S ∆*niuD* | Unmarked deletion mutant, Strepto^R^ | This work |
| B128-S *∆niuB* | B128-S ∆*niuB1 ∆niuB2*, unmarked deletion mutant, Strepto^R^ | This work |
| B128-S ∆*nixA*::*kan* | Km^R^, Strepto^R^ | This work |
| B128-S ∆*nixA*::*kan* ∆*niuD* | Unmarked deletion mutant, Km^R^, Strepto^R^ | This work |
| B128-S ∆*nixA*::*kan* ∆*niuB* | Unmarked deletion mutant, Km^R^, Strepto^R^ | This work |
| B128-S ∆*nixA*::*kan* ∆*niuD* p-*niuDE* | Km^R^, Strepto^R^, *niuDE* on the chromosome (pIRC), Cm^R^ | This work |
| B128-S ∆*nixA*::*kan* ∆*niuB* p-*niuB1* | Km^R^, Strepto^R^, *niuB1* on the chromosome (pIRC), Cm^R^ | This work |
| B128-S ∆*nixA*::*kan* ∆*niuB* p-*niuB2* | Km^R^, Strepto^R^, *niuB2* on the chromosome(pIRC), Cm^R^ | This work |
| B128-S ∆*nixA*::*kan* ∆*niuB* p-*niuB1* | Km^R^, Strepto^R^, *niuB1* on a plasmid (pILL2157), Cm^R^ | This work |
| B128-S ∆*nixA*::*kan* ∆*niuB* p-*niuB2* | Km^R^, Strepto^R^, *niuB2* on plasmid (pILL2157), Cm^R^ | This work |
| B128-S ∆*nikR* | Km^R^, Strepto^R^ | This work |
|  |  |  |
| G27 | Sequenced parental strain | [50] |
|  |  |  |
| SS1 | Sequenced parental strain | [72] |
| SS1-S | SS1 with streptomycin resistance (point mutation) | This work |
| SS1-S ∆*niuD* | Unmarked deletion mutant, Strepto^R^ | This work |
| SS1-S *∆niuB* | SS1-S ∆*niuB1 ∆niuB2*, unmarked deletion mutant, Strepto^R^ | This work |
| SS1-S ∆*nixA*::*kan* | Km^R^, Strepto^R^ | This work |
| SS1-S ∆*nixA*::*kan* ∆*niuD* | Unmarked deletion mutant, Km^R^, Strepto^R^ | This work |
| SS1-S ∆*nixA*::*kan* ∆*niuB* | Unmarked ∆*niuB1 ∆niuB2* deletion mutant, Km^R^, Strepto^R^ | This work |
| SS1-S ∆*niuD* + p-*niuDE* | Unmarked deletion mutant, Strepto^R^, *niuDE* on the chromosome Cm^R^ | This work |
| SS1-S *∆niuB* + p-*niuB1* | unmarked deletion mutant, Strepto^R^, *niuB1* on the chromosome, Cm^R^ | This work |
| SS1-S *∆niuB* + p-*niuB2* | unmarked deletion mutant, Strepto^R^, *niuB2* on the chromosome, Cm^R^ | This work |
|  |  |  |
| 26695 | Sequenced parental strain | [73] |
|  | | |
| **Plasmids** | **Relevant characteristics** | **Reference** |
| *pGEM-T* | Cloning vector | Novagen |
| *pIRCureI* | Cloning vector | [22] |
