# Supplementary material for: Characterization in Helicobacter pylori of a Nickel Transporter Essential for Colonization That Was Acquired during Evolution by Gastric Helicobacter Species
Source: PLoS Pathog. 2016 Dec 6;12(12):e1006018. doi: 10.1371/journal.ppat.1006018 (PMC5140060; doi:10.1371/journal.ppat.1006018)
Supplement: S2 Table — (DOCX) [file ppat.1006018.s010.docx]

**Supplementary S2 Table: oligonucleotides used in this study**

| Construction of unmarked deletions |  | Restriction site of the primer |
| --- | --- | --- |
| niuD-flankA1 | GGTTATGGGGTGTTTGGGAGCG |  |
| niuD-flankA2 | AAA**CTCGAG**GCAAGCTAATGCAATATGATAGG | *Xho*I |
| niuD-flankB1 | AAA**GGATCC**AGAACTAGGGGGGTGTGATG | *Bam*HI |
| niuD-flankB2 | CTAACGCGCTCGTAGGCTCATCTAGTAA |  |
| niuB-flankA1 | CCATGCAAAATGCACTTGTTCG |  |
| niuB-flankA2 | AAA**CTCGAG**AAGCGAGCGATTAGCATAAC | *Xho*I |
| niuB-flankB1 | AAA**GGATCC**TTTTAGCCCTTCGTGTATCG | *Bam*HI |
| niuB-flankB2 | CAGTAGTGTTTGTGGCATTAGC |  |
| Hpn-flankA1 | AAA**GAATTC**GGATATTGAAGCGGCTTGCG | *Eco*RI |
| Hpn-flankA2 | AAA**GGATCC**GTGCCATGATGACTCCTTTG | *Bam*HI |
| Hpn-flankB1 | AAA**GGATCC**GTAATATCGGTGTGGCTAGG | *Bam*HI |
| Hpn-flankB2 | AAA**GAATTC**ATTAAAGCCGTTTAAGATTGTGC | *Bam*HI |
| difHrpsLcat-1 | AAA**CTCGAG**ATTTAAAAGTTTGAAAAG | *Xho*I |
| difHrpsLcat-2 | AAA**GGATCC**ATCGATCATTTAGTTATG | *Bam*HI |
|  |  |  |
| Cloning in pILL2157 |  |  |
| niuDpil-UP | AAA**CATATG**TGTATCAAATCCTCCCGAGTAG | *Nde*I |
| niuDpil-DO | AAA**GATATC**ACTTCTAAGACCATCACACC | *Eco*RV |
| niuDEpil-DO | AAA**GATATC**CTACAACGCATACACGACAAG *Eco*RV |  |
| niuB1pil-UP | AAA**CATATG**TGTTATAATCGCTTCATAAATC | *Nde*I |
| niuB1pil-DO | AAA**GGATCC**CCTAATGCGATACACGAAAGGC | *Bam*HI |
| niuB2pil-UP | AAA**CATATG**ATAATCGCTTTATAAATCATAC | *Nde*I |
| niuB2pil-DO | AAA**GGATCC**CCTAGCGCGATACACGAAGGGC | *Bam*HI |
|  |  |  |
| Cloning in pIRC(P*_ureI_*) |  |  |
| niuDpIR-UP | AAA**GATATC**TGTATCAAATCCTCCCGAGTAG | *Eco*RV |
| niuDpIR-DO | AAA**CCCGGG**ACTTCTAAGACCATCACACC | *Sma*I |
| niuDEpIR-DO | AAA**CCCGGG**CTACAACGCATACACGACAAG | *Sma*I |
| niuB1pIR-UP | AAA**GATATC**TGTTATAATCGCTTCATAAATC | *Eco*RV |
| niuB1pIR-DO | AAA**CCCGGG**CCTAATGCGATACACGAAAGGC | *Sma*I |
| niuB2pIR-UP | AAA**GATATC**ATAATCGCTTTATAAATCATAC | *Eco*RV |
| niuB2pIR-DO | AAA**CCCGGG**CCTAGCGCGATACACGAAGGGC | *Sma*I |
|  |  |  |
| Construction of the P*_fecA3_*::*lacZ* transcriptional fusion |  |  |
| PfecA3-UP | AAA**AGATCT**ATGCAAGCTTATTGTGTGTC | *Bgl*II |
| PfecA3-DO | AAA**ACTAGT**CATATGCTTTTCCTTCCAAACTCCTTGCGTTTTGAG | *Spe*I |
